# Ultrasound-Assisted Synthesis and Biological Profiling of 1,3,5-Triazine Derivatives with Antiproliferative Activity in Triple-Negative Breast Cancer

**DOI:** 10.3390/cimb48030319

**Published:** 2026-03-17

**Authors:** Natalia Bosak, Anna Karolina Drabczyk, Jolanta Jaśkowska, Martyna Stachowicz-Suhs, Beata Filip-Psurska, Anna Boguszewska-Czubara, Katarzyna Ewa Greber, Krzesimir Ciura, Damian Kułaga

**Affiliations:** 1 Department of Organic Chemistry and Technology, Faculty of Chemical Engineering and Technology, Cracow University of Technology, 24 Warszawska Street, 31-155 Cracow, Poland; natalia.bosak54@student.pk.edu.pl (N.B.); anna.drabczyk@pk.edu.pl (A.K.D.); jolanta.jaskowska@pk.edu.pl (J.J.); 2 Department of Experimental Oncology, Hirszfeld Institute of Immunology and Experimental Therapy, Polish Academy of Sciences, 12 Weigla Street, 53-114 Wroclaw, Poland; 3 Innate Immunity Research Group, Life Sciences and Biotechnology Center, Łukasiewicz Research Network-PORT Polish Center for Technology Development, 147 Stabłowicka Street, 54-066 Wrocław, Poland; martyna.stachowicz-suhs@port.lukasiewicz.gov.pl; 4 Laboratory of Experimental Anticancer Therapy, Hirszfeld Institute of Immunology and Experimental Therapy, Polish Academy of Sciences, 12 Weigla Street, 53-114 Wroclaw, Poland; beata.filip-psurska@hirszfeld.pl; 5 Department of Medical Chemistry, Medical University of Lublin, 4a Chodźki Street, 20-093 Lublin, Poland; anna.boguszewska-czubara@umlub.pl; 6 Department of Physical Chemistry, Faculty of Pharmacy, Medical University of Gdansk, 107 General Jozef Haller Avenue, 80-416 Gdansk, Poland; katarzyna.greber@gumed.edu.pl (K.E.G.); krzesimir.ciura@gumed.edu.pl (K.C.)

**Keywords:** triple-negative breast cancer, 1,3,5-triazine, sonochemistry, ultrasound-assisted synthesis, green chemistry

## Abstract

Triple-negative breast cancer (TNBC) remains one of the most aggressive breast cancer subtypes and is associated with limited therapeutic options, underscoring the urgent need for novel treatment strategies. In this study, a library of seventeen 1,3,5-triazine derivatives potentially targeting TNBC was developed using an activity-based approach. Compounds were synthesized via an ultrasound-assisted protocol, providing an efficient and environmentally friendly methodology. The synthesized library was evaluated in vitro against the human TNBC cell lines MDA-MB-468, MDA-MB-231, and Hs578T, as well as the non-tumorigenic epithelial cell line MCF10A. Compounds **9** and **17** exhibited the most promising antiproliferative activity against TNBC cell lines (MDA-MB-468: IC_50_ = 36.62 µM for **9** and 38.29 µM for **17**; MDA-MB-231: IC_50_ = 37.32 µM for **9** and 32.86 µM for **17**; Hs578T: IC_50_ = 57.26 µM for **9** and 34.87 µM for **17**), while maintaining acceptable selectivity toward non-cancerous cells. The lead compounds were further assessed in vivo using a *Danio rerio* model to evaluate general toxicity and cardiotoxicity. In addition, ADME parameters were predicted for all compounds using biomimetic chromatography. Overall, compounds **9** and **17** emerged as promising small-molecule candidates for TNBC treatment, requiring further toxicological evaluation in more human-relevant in vivo models.

## 1. Introduction

Triple-negative breast cancer (TNBC) is one of the most aggressive subtypes of breast cancer, accounting for approximately 10–15% of all cases. It is characterized by the lack of estrogen receptor (ER) and progesterone receptor (PR) expression, as well as the absence of human epidermal growth factor receptor 2 (HER2) overexpression [1]. The absence of these receptors eliminates the main therapeutic targets, making hormone therapy and HER2-directed treatments used in other breast cancer subtypes ineffective [1,2]. Consequently, for many years chemotherapy was the only available systemic treatment option for TNBC [2].

Patients with TNBC tend to experience more aggressive disease progression than those with other breast cancer subtypes [1]. Studies have shown that survival rate in TNBC is significantly lower than in receptor-positive breast cancers across all risk groups, including age, race, and tumor size [3]. Moreover, TNBC is associated with higher relapse rates, increased probability of metastasis and high proliferation index [1,4]. Recurrence in TNBC occurs in about 33.9% of patients, compared with 20.4% in other breast cancer subtypes. It typically begins as a locoregional relapse before progressing to distant organs [3]. TNBC also exhibits a metastatic pattern: metastases more frequently affect visceral organs, soft tissues and the central nervous system, particularly the lungs, liver and brain [1,3]. In contrast, bone recurrence is less common than in hormone receptor–positive disease [3]. Survival in metastatic TNBC is approximately 12 months, which is shorter than for other breast cancer subtypes [4], and the median overall survival for TNBC patients rarely exceeds 2 years [5]. Therefore, there is an urgent need to develop more effective therapeutic strategies for this disease.

Currently, chemotherapy remains the standard treatment for TNBC, particularly for tumors measuring 2 cm or larger, or involving lymph nodes [2]. Neoadjuvant chemotherapy, therapy administered prior to surgery, increases the likelihood of achieving a pathological complete response (pCR) at the time of surgery, which can lead to improvement in disease-free survival (DFS) [6]. Moreover, studies suggest that TNBC may be more responsive to chemotherapy than other breast cancer subtypes. The most commonly used chemotherapeutic agents for TNBC include anthracyclines such as doxorubicin (Adriamycin) and taxanes such as paclitaxel (Taxol). Additionally, immunotherapy with atezolizumab (Tecentriq) or pembrolizumab (Keytruda) can be combined with chemotherapy to further improve pCR rates and potentially delay disease progression [2,3].

**Figure 1:**
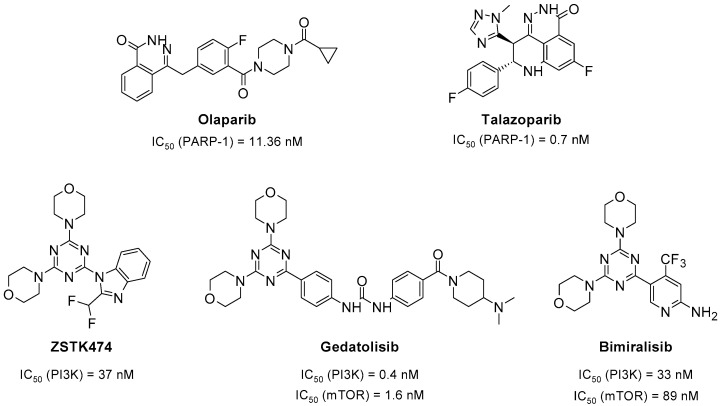
Examples of 2,3,4,5-tetrahydropyridazine derivatives used in the treatment of TNBC (olaparib, talazoparib) and 1,3,5-triazine derivatives in the clinical research phase (ZSTK474, gedatolisib, bimiralisib) [7,10,11].

When it comes to the therapy for triple-negative breast cancer (TNBC), one of the most significant advances has been the implementation of poly(ADP-ribose) polymerase (PARP) inhibitors, targeting tumors with impaired DNA damage repair via the homologous recombination mechanism, particularly in patients with germline BRCA1/2 mutations [2,3]. Within the PARP inhibitor class, two compounds have gained approval for use in TNBC treatment: olaparib (Lynparza) and talazoparib (Talzenna) (Figure 1), both of which are classified as 2,3,4,5-tetrahydropyridazine derivatives [3]. Olaparib exhibits activity against PARP-1, PARP-2, and PARP-3 [3,4,7], and beyond TNBC, it is also utilized in selected gynecological malignancies (including epithelial ovarian cancer, fallopian tube cancer and primary peritoneal cancer) [8]. Talazoparib, on the other hand, is characterized by a dual mechanism of action—it inhibits the catalytic activity of PARP and induces “PARP trapping”, which enhances the cytotoxic effect in DNA repair-deficient cells [3,9,10]. Although both drugs are generally well-tolerated, the most frequently observed adverse events include constitutional and hematological symptoms such as fatigue, nausea and anemia, while thrombocytopenia and neutropenia have also been reported with talazoparib [4]. Even though PARP inhibitors remain primarily a monotherapy, combination therapy strategies involving chemotherapy and targeted therapies are being intensively developed [3].

In parallel, the inhibition of the PI3K/AKT/mTOR pathway has emerged as a promising strategy in TNBC therapy, as its dysregulation promotes tumor growth, cell survival, and disease progression [11,12,13]. In recent years, 1,3,5-triazine-based small molecules have attracted attention due to their anticancer potential across multiple tumor types, with many derivatives acting as potent PI3K isoform or dual PI3K/mTOR inhibitors, supporting the exploration of this scaffold for TNBC treatment [14,15,16].

Among the triazine compounds clinically evaluated in TNBC, the pan-PI3K inhibitor ZSTK474 and the dual PI3K/mTOR inhibitors gedatolisib and bimiralisib (Figure 1) stand out [11,17]. Gedatolisib (PF-05212384, PKI-587) demonstrates PI3K/mTOR inhibitory activity with an acceptable safety profile in oncology trials [18,19], whereas bimiralisib (PQR309) has shown anticancer activity in advanced solid tumors and is currently under clinical evaluation in breast cancer, including TNBC [20,21]. ZSTK474, an oral Class I PI3K inhibitor, has demonstrated anticancer activity in preclinical models and early-phase clinical trials in solid tumors, though clinical data regarding TNBC remain limited [20,22,23].

In the context of synthesizing 1,3,5-triazine derivatives, cyanuric chloride (2,4,6-trichloro-1,3,5-triazine) remains the most widely utilized starting material [15]. Due to the predictable, temperature-dependent reactivity of its electrophilic centers, the chlorine atoms undergo stepwise nucleophilic aromatic substitution. Consequently, the first substitution typically occurs at 0 °C and the second at room temperature, whereas the third substitution requires a significantly higher energy input [24]. This final stage often necessitates heating under reflux in organic solvents like DMF, ACN, or 1,4-dioxane [25]. However, given the strategic shift toward Green Chemistry and sustainable Active Pharmaceutical Ingredients (API) production in the European Union (EU), traditional methods characterized by high energy consumption and hazardous solvents are increasingly unfavorable [26]. A robust alternative is ultrasound-assisted synthesis. Acoustic cavitation, which occurs by the formation, growth and collapse of bubbles in a liquid, generates localized ‘hotspots’ with extreme temperatures and pressures, providing the necessary activation energy to facilitate the third nucleophilic aromatic substitution under mild conditions [27].

Our previous findings demonstrate that ultrasound-assisted synthesis is a robust and versatile approach for constructing the derivatives with 1,3,5-triazine scaffold. Furthermore, while conventional ultrasonic baths can be successfully employed to obtain these derivatives, the use of a dedicated ultrasonic reactor proved significantly more efficient, facilitating a twelve-fold reduction in reaction time. Notably, the incorporation of tetrabutylammonium bromide (TBAB) as a phase transfer catalyst (PTC) in an aqueous medium with sodium carbonate achieved a peak efficiency of 98%. The impact of ultrasonic activation was conclusive. While synthesizing the same derivative without ultrasonic assistance required 20 min of stirring and resulted in a limited yield of 56%, the optimized sonochemical conditions facilitated a ten-fold reduction in reaction time. Specifically, the yield increased to 88% after only 2 min of ultrasonic irradiation. Extending the reaction time to 5 min further increased the yield to 97%. Beyond the speed and efficiency, this methodology offers superior regioselectivity and ease of purification. Following a simple pH-dependent work-up, the final product was isolated with a 93% yield, requiring no further chromatographic purification. This represents a significant advancement in the sustainable and cost-effective synthesis of biologically active triazine scaffolds [14].

Importantly, ultrasound-assisted synthesis is not limited to 1,3,5-triazine derivatives. Its applicability has been demonstrated in the synthesis of pharmaceutically relevant compounds, such as olanzapine and quetiapine, antipsychotic agents that are dibenzazepine derivatives [28], as well as phenytoin, an anti-seizure agent [29]. Additionally, several anticancer drug candidates have been studied using this approach, including 3,5-disubstituted sulphonamide isoxazoline derivatives assessed for their antineoplastic activity against model leukemia cell lines [30], 2-amino-4,6-disubstituted nicotinonitrile derivatives evaluated as potential SIRT1 inhibitors [31], and hydrazine carboxamides tested against multiple cancer cell lines [32]. Moreover, it is worth noting that sonochemical methodologies have broader applications in pharmaceutical research beyond optimizing compound synthesis. For instance, ultrasound-assisted extraction has been used to isolate polysaccharides from *Ginkgo biloba*, demonstrating anti-inflammatory activity [33]. Other examples include ultrasound-assisted upconversion photodynamic therapy, enabling the elimination of fungal biofilm infections [34], and ultrasonic complexation of ibuprofen with silver, which could activate the drug and enhance its efficacy in microwave chemotherapy [35]. These findings highlight that ultrasound-assisted approaches have diverse applications, not only in drug candidate synthesis but more broadly in pharmaceutical research.

Since 1,3,5-triazine derivatives have emerged as promising drug candidates for TNBC treatment [11], and no FDA-approved therapeutics based on this scaffold are currently available, there is significant potential for further development in this area. Given the lack of a distinct molecular target in TNBC, the design of such compounds can be guided by activity-based rather than target-based approaches. In this study, a series of 2-amino-1,3,5-triazine derivatives was developed with the intention of identifying new agents active against TNBC. Compounds were synthesized through a three-step protocol, with the final step performed using a sonochemistry method, which provides a more sustainable and time-efficient alternative to conventional synthetic procedures [36]. The final derivatives were evaluated in vitro against the human TNBC cell lines MDA-MB-468, MDA-MB-231, and Hs578T, as well as the non-tumorigenic epithelial cell line MCF10A. The most promising candidates were further assessed in vivo using the *Danio rerio* model to evaluate general toxicity and cardiotoxicity. Finally, biomimetic chromatography was applied to predict ADME parameters, an essential component in identifying drug candidates with a higher likelihood of successful progression.

## 2. Materials and Methods

### 2.1. Chemistry

#### 2.1.1. General

All reagents, TLC plates, and silica gel were obtained from commercial suppliers (Chemat, Gdańsk, Poland). Purification-grade solvents were purchased from Krakchemia, Kraków, Poland, whereas LC–MS solvents were supplied by Witko, Łódź, Poland. Analytical thin-layer chromatography was carried out on aluminum-backed silica gel plates (0.2 mm, 60 F254, Merck, Darmstadt, Germany), and chromatograms were visualized under UV light at 254 nm. Melting points were measured using a Boetius melting point apparatus. ^1^H-NMR spectra were recorded on a Bruker spectrometer operating at 400 MHz, with tetramethylsilane (TMS) used as an internal chemical shift reference (δ = 0 ppm). MestReNova 15.01 was used for raw NMR data processing. The final compounds were prepared for analysis in DMSO-d_6_ or CD_3_OD. NMR spectra are available in Supplementary Materials. For compounds **1**–**13**, **15**–**17**, and intermediates **23**–**24**, UPLC-MS/MS analyses were performed using a Waters ACQUITY UPLC system coupled to a Waters TQD tandem quadrupole mass spectrometer equipped with an electrospray ionization (ESI) source. Separation was achieved on an Acquity UPLC BEH C18 column (2.1 × 100 mm, 1.7 μm) fitted with a VanGuard BEH C18 precolumn (2.1 × 5 mm, 1.7 μm). The column temperature was maintained at 40 °C. Gradient elution was performed at a flow rate of 0.3 mL·min^−1^, decreasing eluent D from 95% to 0% over 10 min. Eluent D consisted of water with 0.1% formic acid, while eluent E contained acetonitrile with 0.1% formic acid. Chromatographic data were collected using a Waters photodiode array detector. UV spectra were recorded between 200 and 700 nm at a resolution of 1.2 nm and a sampling rate of 20 points per second. Mass spectrometric parameters were set as follows: source temperature 150 °C, desolvation temperature 350 °C, desolvation gas flow 600 L·h^−1^, cone gas flow 100 L·h^−1^, capillary voltage 3.0 kV, and cone voltage 30 V. Nitrogen served as both nebulizing and desolvation gas. Data were acquired in scan mode over an *m*/*z* range of 50–1000 with a scan time of 0.5 s. Instrument control and data processing were performed using MassLynx software (version 4.1). High-resolution LC–MS measurements for compounds **1**–**17** were conducted on a Shimadzu Nexera LC-40 XS system equipped with a PDA detector (SPD-M40) and an LCMS-9030 quadrupole time-of-flight (QTOF) mass spectrometer. Chromatographic separation was carried out on a Shim-pack Scepter C18 column (3 μm, 100 × 2.1 mm). The mobile phase consisted of solvent A (water containing 0.1% formic acid) and solvent B (acetonitrile containing 0.1% formic acid) applied under gradient conditions: 5% B at 0 min, 95% B at 6 min, maintained until 7 min, returned to 5% B at 7.1 min, with a total run time of 10 min. The flow rate was set at 0.4 mL·min^−1^. UV-Vis spectra were recorded between 190 and 700 nm. Mass spectra were collected in positive ESI mode across an *m*/*z* range of 100–1000, with a scan speed of 15,000 u·s^−1^ and event time of 0.2 s. QTOF operating conditions included heating gas flow 10 L·min^−1^, interface temperature 300 °C, desolvation line temperature 250 °C, heat block temperature 400 °C, drying gas flow 10 L·min^−1^, and interface voltage +4.5 kV. External mass calibration was performed using a sodium iodide solution in methanol to ensure high mass accuracy. Data acquisition and processing were carried out using LabSolutions software (version 5.128 SP1). Ultrasound-assisted synthesis of the final compounds was performed using a Sonicator VCX 130 PB ultrasonic processor operating at 70% amplitude. HRMS spectra are available in Supplementary Materials.

#### 2.1.2. General Procedure for the Synthesis of Intermediates 23–24

A round-bottom flask was charged with cyanuric chloride **18** (3.2 g, 17.35 mmol) and dissolved in 30 mL THF. The mixture was stirred for a few minutes before a solution of aniline **19** (1.47 g, 15.77 mmol) or 4-chloroaniline 20 (2.01 g, 15.77 mmol) in 10 mL of THF was added dropwise while maintaining the reaction temperature at 0–5 °C. The mixture was stirred for a few minutes at 0–5 °C and then allowed to warm up to room temperature. Upon completion of the reaction, the solvent was evaporated to dryness under reduced pressure and the crude product was macerated with cold methanol. The resulting solids **21** or **22** were filtered off, evaporated to dryness and used in the next step without further purification. Next, Intermediate **21** (3 g, 12.44 mmol) or **22** (3 g, 10.89 mmol) was dissolved in 50 mL of THF and 5.5 mL of 25% aqueous ammonia solution was added. The reaction mixture was stirred overnight at room temperature. Upon completion of the reaction, the precipitated solids **23** or **24** were filtered off, evaporated to dryness under reduced pressure and macerated with cold methanol. The resulting solids were collected by filtration, evaporated to dryness and used in the next step without further purification.

6-chloro-N^2^-phenyl-1,3,5-triazine-2,4-diamine (**23**)

White solid (76% yield), m.p.: 211–213 °C. UHPLC-MS analysis t = 0.42 min (99.5% purity), calc. for C_9_H_8_ClN_5_ *m*/*z* = 221.05, found *m*/*z* = 222.1 [M+H]^+^

6-chloro-N^2^-(4-chlorophenyl)-1,3,5-triazine-2,4-diamine (**24**)

Beige solid (88% yield), m.p.: 230–235 °C. UHPLC-MS analysis t = 0.42 min (99.5% purity), calc. for C_9_H_7_Cl_2_N_5_ *m*/*z* = 255.01, found *m*/*z* = 256.06 [M+H]^+^

#### 2.1.3. General Procedure for the Synthesis of Final Compounds 1–17

In total, 1 mmol quantities of intermediate **23** or **24**, potassium carbonate (3 mmol) and TBAB (0.1 mmol) were ground in a mortar and transferred to a sealed tube. Commercially available piperazine derivative (**25**–**33** for set I; **25**–**30** and **33**–**34** for set II) was added (2.5 mmol). Subsequently, 0.5 mL of water was added. The reaction mixture was subjected to ultrasonic irradiation in a Sonicator VCX 130 PB at 70% amplitude in pulsed mode (10 s on/10 s off), with a total effective sonication time of 5 min. Reaction progress was monitored via TLC (hexane: EtOAc 1:1 *v*/*v*). After the completion of reaction, the mixture was cooled down and extracted with dichloromethane and water. Organic layer was dried over MgSO_4_ and concentrated in vacuo. The crude product was purified via column chromatography eluting from 100% chloroform to chloroform: MeOH 95:5 *v*/*v.* Purified compound was dissolved in acetone, then pH was adjusted to 2–3 with 4 M HCl in 1,4-dioxane and cold diethyl ether was added. The precipitating solid was filtered and dried to yield title compounds **1**–**17**.

6-(4-methylpiperazin-1-yl)-N^2^-phenyl-1,3,5-triazine-2,4-diamine hydrochloride (**1**)

Beige solid (83% yield), m.p.: 154–155 °C, ^1^H NMR (400 MHz, CD_3_OD) δ 7.52 (d, J = 7.7 Hz, 2H), 7.46 (t, J = 7.7 Hz, 2H), 7.30 (br-s, 1H), 3.64 (d, J = 10.2 Hz, 2H), 3.51 (t, J = 12.4 Hz, 2H), 3.33 (dt, J = 3.2, 1.6 Hz, 2H), 3.22 (d, J = 10.2 Hz, 2H), 2.97 (s, 3H). UHPLC-MS analysis: t = 2.09 min (100.00% purity), calc. for base C_14_H_19_N_7_ *m*/*z* = 285.17, found *m*/*z* = 286.23 [M+H]^+^; HRMS (ESI) [M+H]^+^ calc. for C_14_H_19_N_7_: 286.17747, found 286.17700

N^2^-phenyl-6-(4-phenylpiperazin-1-yl)-1,3,5-triazine-2,4-diamine hydrochloride (**2**)

Beige solid (87% yield), m.p.: 165–167 °C, ^1^H NMR (400 MHz, CD_3_OD) δ 7.72 (d, J = 8.1 Hz, 2H), 7.61 (t, J = 7.8 Hz, 2H), 7.54 (d, J = 7.3 Hz, 3H), 7.47 (t, J = 7.4 Hz, 2H), 7.30 (br-s, 1H), 4.4 (brs, 4H), 3.79 (t, J = 5.1 Hz, 4H). UHPLC-MS analysis: t = 5.87 min (98.59% purity), calc. for base C_19_H_21_N_7_ *m*/*z* = 347.19, found *m*/*z* = 348.31 [M+H]^+^; HRMS (ESI) [M+H]^+^ calc. for C_19_H_21_N_7_: 348.19312, found 348.19297

6-(4-(2-chlorophenyl)piperazin-1-yl)-N^2^-phenyl-1,3,5-triazine-2,4-diamine hydrochloride (**3**)

Beige solid (69% yield), m.p.: 181–183 °C, ^1^H NMR (400 MHz, CD_3_OD) δ 7.53 (dd, J = 12.5, 7.9 Hz, 3H), 7.45 (t, J = 7.8 Hz, 2H), 7.39 (d, J = 4.0 Hz, 2H), 7.31–7.20 (m, 2H), 4.19 (br-s, 4H), 3.38 (t, J = 5.1 Hz, 4H). UHPLC-MS analysis: t = 6.64 min (99.64% purity), calc. for base C_19_H_20_ClN_7_ *m*/*z* = 381.15, found *m*/*z* = 382.27 [M+H]^+^; HRMS (ESI) [M+H]^+^ calc. for C_19_H_20_ClN_7_: 382.15415, found 382.15397

6-(4-(3-chlorophenyl)piperazin-1-yl)-N^2^-phenyl-1,3,5-triazine-2,4-diamine hydrochloride (**4**)

Beige solid (72% yield), m.p.: 170–171 °C, ^1^H NMR (400 MHz, CD_3_OD) δ 7.54 (d, J = 7.8 Hz, 2H), 7.46 (dt, J = 11.6, 3.8 Hz, 3H), 7.30–7.25 (m, 3H), 7.13 (t, J = 7.6 Hz, 1H), 4.12 (brs, 4H), 3.22 (t, J = 5.0 Hz, 4H)). UHPLC-MS analysis: t = 6.60 min (99.66% purity), calc. for base C_19_H_20_ClN_7_ *m*/*z* = 381.15, found *m*/*z* = 382.20 [M+H]^+^; HRMS (ESI) [M+H]^+^ calc. for C_19_H_20_ClN_7_: 382.15415, found 382.15390

6-(4-(4-chlorophenyl)piperazin-1-yl)-N^2^-phenyl-1,3,5-triazine-2,4-diamine hydrochloride (**5**)

Beige solid (68% yield), m.p.: 162–164 °C, ^1^H NMR (400 MHz, CD_3_OD) δ 7.63 (d, J = 9.0 Hz, 2H), 7.57–7.51 (m, 4H), 7.46 (t, J = 7.8 Hz, 2H), 7.29 (s, 1H), 4.33 (s, 2H), 3.69 (dd, J = 8.3, 3.1 Hz, 4H), 3.33 (dt, J = 3.3, 1.6 Hz, 2H). UHPLC-MS analysis: t = 6.71 min (91.75% purity), calc. for base C_19_H_20_ClN_7_ *m*/*z* = 381.15, found *m*/*z* = 382.20 [M+H]^+^; HRMS (ESI) [M+H]^+^ calc. for C_19_H_20_ClN_7_: 382.15415, found 382.15390

6-(4-(4-methoxyphenyl)piperazin-1-yl)-N^2^-phenyl-1,3,5-triazine-2,4-diamine hydrochloride (**6**)

Beige solid (90% yield), m.p.: 189–191 °C, ^1^H NMR (400 MHz, DMSO) δ 10.53 (s, 1H), 8.16 (s, 2H), 7.59 (d, J = 8.0 Hz, 1H), 7.39 (t, J = 7.9 Hz, 1H), 7.31 (brs, 1H), 7.17 (t, J = 7.4 Hz, 1H), 7.03–6.92 (m, 3H), 6.91–6.83 (m, 2H), 3.70 (s, 3H), 3.23 (d, J = 5.2 Hz, 8H). UHPLC-MS analysis: t = 5.29 min (97.19% purity), calc. for base C_20_H_23_N_7_O *m*/*z* = 377.20, found *m*/*z* = 378.28 [M+H]^+^; HRMS (ESI) [M+H]^+^ calc. for C_20_H_23_ClN_7_O: 378.20368, found 378.20300

6-(4-(2-methoxyphenyl)piperazin-1-yl)-N^2^-phenyl-1,3,5-triazine-2,4-diamine hydrochloride (**7**)

Beige solid (82% yield), m.p.: 159–162 °C, ^1^H NMR (400 MHz, CD_3_OD) δ 7.73 (dd, J = 8.2, 1.2 Hz, 1H), 7.61–7.51 (m, 3H), 7.46 (t, J = 7.5 Hz, 2H), 7.35 (dd, J = 8.4, 0.9 Hz, 1H), 7.30 (s, 1H), 7.23–7.16 (m, 1H), 4.41 (brs, 4H), 4.08 (s, 3H), 3.86 (t, J = 5.2 Hz, 4H). UHPLC-MS analysis: t = 5.63 min (97.69% purity), calc. for base C_20_H_23_N_7_O *m*/*z* = 377.20, found *m*/*z* = 378.28 [M+H]^+^; HRMS (ESI) [M+H]^+^ calc. for C_20_H_23_N_7_O: 378.20368, found 378.20334

6-(4-benzylpiperazin-1-yl)-N^2^-phenyl-1,3,5-triazine-2,4-diamine hydrochloride (**8**)

Beige solid (89% yield), m.p.: 173–175 °C, ^1^H NMR (400 MHz, CD_3_OD) δ 7.62 (dd, J = 6.5, 2.9 Hz, 2H), 7.56–7.42 (m, 7H), 7.30 (s, 1H), 4.44 (s, 2H), 3.64–3.43 (m, 4H), 3.33–3.15 (m, 4H). UHPLC-MS analysis: t = 3.61 min (97.23% purity), calc. for base C_20_H_23_N_7_ *m*/*z* = 361.20, found *m*/*z* = 362.26 [M+H]*^+^*; HRMS (ESI) [M+H]^+^ calc. for C_20_H_23_N_7_: 362.20877, found 362.20844

6-(4-(benzo[d]isothiazol-3-yl)piperazin-1-yl)-N^2^-phenyl-1,3,5-triazine-2,4-diamine hydrochloride (**9**)

Beige solid (67% yield), m.p.: 190–193 °C, ^1^H NMR (400 MHz, CD_3_OD) δ 8.13 (d, J = 8.1 Hz, 1H), 7.96 (d, J = 8.1 Hz, 1H), 7.63–7.41 (m, 6H), 7.28 (t, J = 6.8 Hz, 1H), 4.15 (brs, 4H), 3.67–3.62 (m, 4H). UHPLC-MS analysis: t = 6.28 min (90.87% purity), calc. for base C_20_H_20_N_8_S *m*/*z* = 404.15, found *m*/*z* = 405.26 [M+H]*^+^*; HRMS (ESI) [M+H]^+^ calc. for C_20_H_20_N_8_S: 405.16044, found 405.16020

N^2^-(4-chlorophenyl)-6-(4-methylpiperazin-1-yl)-1,3,5-triazine-2,4-diamine hydrochloride (**10**)

Beige solid (85% yield), m.p.: 175–177 °C, ^1^H NMR (400 MHz, CD_3_OD) δ 7.53 (d, J = 8.8 Hz, 2H), 7.45 (d, J = 8.8 Hz, 2H), 3.69–3.60 (m, 2H), 3.50 (t, J = 12.8 Hz, 2H), 3.33 (dt, J = 3.3, 1.6 Hz, 2H), 3.20 (t, J = 11.1 Hz, 2H), 2.97 (s, 3H). UHPLC-MS analysis: t = 3.13 min (100.00% purity), calc. for base C_14_H_18_ClN_7_ *m*/*z* = 319.13, found *m*/*z* = 320.26 [M+H]*^+^*; HRMS (ESI) [M+H]^+^ calc. for C_14_H_18_ClN_7_: 320.13850, found 320.13838

N^2^-(4-chlorophenyl)-6-(4-phenylpiperazin-1-yl)-1,3,5-triazine-2,4-diamine hydrochloride (**11**)

Beige solid (78% yield), m.p.: 157–160 °C, ^1^H NMR (400 MHz, CD_3_OD) δ 7.56 (d, J = 8.8 Hz, 2H), 7.31–7.36 (m, 4H), 7.36 (d, J = 8.3 Hz, 1H), 7.3 (s, 1H), 7.23 (d, J = 7.3 Hz, 1H), 4.13 (brs, 4H), 3.72–3.67 (m, 4H). UHPLC-MS analysis: t = 6.71 min (95.72% purity), calc. for base C_19_H_20_ClN_7_ *m*/*z* = 381.15, found *m*/*z* = 382.27 [M+H]*^+^*; HRMS (ESI) [M+H]^+^ calc. for C_19_H_20_ClN_7_: 382.15415, found 382.15404

N^2^-(4-chlorophenyl)-6-(4-(2-chlorophenyl)piperazin-1-yl)-1,3,5-triazine-2,4-diamine hydrochloride (**12**)

Beige solid (70% yield), m.p.: 178–180 °C, ^1^H NMR (400 MHz, CD_3_OD) δ 7.57 (d, J = 8.8 Hz, 2H), 7.49–7.40 (m, 3H), 7.38–7.31 (m, 1H), 7.29–7.25 (m, 1H), 7.14 (td, J = 7.9, 1.5 Hz, 1H), 4.12 (brs, 4H), 3.27–3.23 (m, 4H). UHPLC-MS analysis: t = 7.43 min (98.02% purity), calc. for base C_19_H_19_Cl_2_N_7_ *m*/*z* = 415.11, found *m*/*z* = 416.16 [M+H]*^+^*; HRMS (ESI) [M+H]^+^ calc. for C_19_H_19_Cl_2_N_7_: 416.11518, found 416.11470

N^2^-(4-chlorophenyl)-6-(4-(3-chlorophenyl)piperazin-1-yl)-1,3,5-triazine-2,4-diamine hydrochloride (**13**)

Beige solid (72% yield), m.p.: 183–185 °C, ^1^H NMR (400 MHz, CD_3_OD) δ 7.56 (d, J = 8.8 Hz, 2H), 7.49–7.40 (m, 4H), 7.36 (d, J = 8.3 Hz, 1H), 7.23 (d, J = 7.3 Hz, 1H), 4.22 (s, 4H), 3.61–3.55 (m, 4H). UHPLC-MS analysis: t = 7.53 min (98.36% purity), calc. for base C_19_H_19_Cl_2_N_7_ *m*/*z* = 415.11, found *m*/*z* = 416.23 [M+H]*^+^*; HRMS (ESI) [M+H]^+^ calc. for C_19_H_19_Cl_2_N_7_: 416.11518, found 416.11500

N^2^-(4-chlorophenyl)-6-(4-(4-chlorophenyl)piperazin-1-yl)-1,3,5-triazine-2,4-diamine hydrochloride (**14**)

Beige solid (80% yield), m.p.: 194–196 °C, ^1^H NMR (400 MHz, MeOD) δ 7.58–7.53 (m, 2H), 7.51–7.41 (m, 6H), 4.26 (brs, 3H), 3.87 (t, J = 5.0 Hz, 1H), 3.75 (t, J = 4.9 Hz, 1H), 3.61 (t, J = 5.3 Hz, 3H). LC-HRMS analysis: t = 5.26 min (90.21% purity), calc. for base C_19_H_19_Cl_2_N_7_ *m*/*z* = 415.1152, found *m*/*z* = 416.1148 [M+H]^+^; HRMS (ESI) [M+H]^+^ calc. for C_19_H_19_Cl_2_N_7_: 416.11518, found 416.11500

N^2^-(4-chlorophenyl)-6-(4-(4-methoxyphenyl)piperazin-1-yl)-1,3,5-triazine-2,4-diamine hydrochloride (**15**)

Beige solid (78% yield), m.p.: 184–185 °C, ^1^H NMR (400 MHz, CD_3_OD) δ 7.70 (d, J = 7.8 Hz, 1H), 7.55 (t, J = 7.5 Hz, 3H), 7.45 (d, J = 8.6 Hz, 2H), 7.33 (d, J = 7.8 Hz, 1H), 7.18 (t, J = 7.6 Hz, 1H), 4.38 (s, 4H), 3.83 (t, J = 4.7 Hz, 4H). UHPLC-MS analysis: t = 6.41 min (97.72% purity), calc. for base C_20_H_22_ClN_7_O *m*/*z* = 411.16, found *m*/*z* = 412.24 [M+H]*^+^*; HRMS (ESI) [M+H]^+^ calc. for C_20_H_22_ClN_7_O: 412.16471, found 412.16401

N^2^-(4-chlorophenyl)-6-(4-(3-methoxyphenyl)piperazin-1-yl)-1,3,5-triazine-2,4-diamine hydrochloride (**16**)

Beige solid (68% yield), m.p.: 173–175 °C, ^1^H NMR (400 MHz, MeOD) δ 7.70 (dd, J = 8.3, 1.4 Hz, 1H), 7.56 (t, J = 7.4 Hz, 3H), 7.45 (d, J = 8.4 Hz, 2H), 7.33 (dd, J = 8.5, 1.2 Hz, 1H), 7.22–7.15 (t, J = 7.9 Hz, 1H), 4.38 (brs, 4H), 4.07 (s, 3H), 3.83 (t, J = 5.1 Hz, 4H). UHPLC-MS analysis: t = 6.25 min (98.25% purity), calc. for base C_20_H_22_ClN_7_O *m*/*z* = 411.16, found *m*/*z* = 412.24 [M+H]*^+^*; HRMS (ESI) [M+H]^+^ calc. for C_20_H_22_ClN_7_O: 412.16471, found 412.16400

6-(4-(benzo[d]isothiazol-3-yl)piperazin-1-yl)-N^2^-(4-chlorophenyl)-1,3,5-triazine-2,4-diamine hydrochloride (**17**)

Beige solid (74% yield), m.p.: 181–183 °C, ^1^H NMR (600 MHz, CD_3_OD) δ 8.09 (dt, J = 8.2, 1.0 Hz, 1H), 7.94 (dt, J = 8.2, 0.9 Hz, 1H), 7.60–7.51 (m, 3H), 7.49–7.40 (m, 3H), 4.12 (t, J = 5.1 Hz, 4H), 3.64–3.60 (m, 4H). UHPLC-MS analysis: t = 7.18 min (98.16% purity), calc. for base C_20_H_19_ClN_8_S *m*/*z* = 438.11, found *m*/*z* = 439.23 [M+H]*^+^*; HRMS (ESI) [M+H]^+^ calc. for C_20_H_19_ClN_8_S: 439.12147, found 439.12100

### 2.2. In Vitro Assay

#### 2.2.1. Cell Culture and Maintenance, Compound Dilution

The human breast cancer cell lines MDA-MB-231 and Hs578T, as well as the non-tumorigenic human breast epithelial cell line MCF10A, were obtained from the American Type Culture Collection (ATCC). Human breast cancer cel line MDA-MB-468 was obtained from German Collection of Microorganisms and Cell Cultures (DSMZ). MDA-MB-231 cells were cultured in RPMI 1640 medium supplemented with 10% fetal bovine serum (FBS), 2 mM L-glutamine and antibiotics: 100 U/mL penicillin and 100 µg/mL streptomycin. MDA-MB-468 cells were maintained in RPMI 1640 medium with GlutaMAX supplement, 20% FBS and antibiotics: 100 U/mL penicillin and 100 µg/mL streptomycin. Hs578T cells were grown in Dulbecco’s Modified Eagle Medium (DMEM; Gibco, Grand Island, NY, USA) supplemented with 10% FBS, 2 mM L-glutamine, 0.1 mL insulin, 100 U/mL penicillin and 100 µg/mL streptomycin. MCF10A cells were cultured in Ham’s F-12 medium (with L-glutamine; Gibco) supplemented with 5% horse serum, epidermal growth factor (EGF; 100 µL per 100 mL medium), insulin (100 µL per 100 mL medium), hydrocortisone (1 mL per 100 mL medium), cholera toxin (0.1 mL per 100 mL medium) and antibiotics (100 U/mL penicillin and 100 µg/mL streptomycin). Cells were incubated at 37 °C in a humidified atmosphere containing 5% CO_2_. Compound dilutions were prepared in RPMI 1640 medium supplemented with 10% FBS, 100 U/mL penicillin, and 100 μg/mL streptomycin.

#### 2.2.2. Evaluation of Antiproliferative Activity (MTT Assay)

Passaged cells were seeded in 96-well plates at a density of 2 × 10^5^ cells/mL in 100 µL of the appropriate growth medium. Cells were incubated at 37 °C in a humidified atmosphere containing 5% CO_2_ for 24 h. After that time, an additional 100 µL of growth medium (growth control) or medium containing the tested compounds was added to each well. Following concentrations were applied: 6.25, 12.5, 25, 50 µM or 12.5, 25, 50, 100 µM. Plates were then incubated for another 24 h under standard culture conditions (37 °C, 5% CO_2_). Cell viability was assessed using the MTT performed as per instructions. Absorbance at 570 nm was measured using Synergy H4 Hybrid Reader. The analysis of measurement results was performed using the Prolab system. IC_50_ values were calculated relative to untreated controls. Experiments were performed in triplicate and repeated 3–4 times.

### 2.3. In Vivo Toxicity

Compound toxicity was evaluated using the zebrafish embryo toxicity assay performed in accordance with OECD Test Guideline 236. Fertilized zebrafish (*Danio rerio*) embryos were initially collected and maintained in Petri dishes filled with E3 medium composed of 5 mM NaCl, 0.33 mM MgCl_2_, 0.33 mM CaCl_2_, and 0.17 mM KCl (pH 7.2). Subsequently, embryos were distributed into 6-well plates at a density of ten embryos per well. Stock solutions of compounds **9** and **17**, together with the reference compound cisplatin, were prepared in dimethyl sulfoxide (DMSO). Working solutions at the required concentration ranges were freshly prepared by diluting stock solutions in E3 medium immediately before application. Test media were renewed daily, and embryos were incubated at 28.5 °C throughout the experiment. Acute toxicity was determined at 96 h post-fertilization (hpf) based on the occurrence of four morphological lethality criteria: egg coagulation, failure of somite formation, absence of tailbud detachment from the yolk sac, and lack of cardiac activity. Heart rate measurements were additionally performed to assess potential cardiotoxic effects. Embryos were examined using a Discovery V8 stereomicroscope (Zeiss). Dose–response relationships were analyzed using GraphPad Prism 8.0.1 by fitting a sigmoidal model to the experimental data, allowing calculation of LC_50_ values for the tested compounds.

### 2.4. Biomimetic Chromatography—Lipophilicity and Drug-Plasma Protein Binding

Lipophilicity and plasma protein binding were determined according to biomimetic chromatographic procedures developed by Valko et al. [37,38] and routinely applied in our laboratory [39,40]. Analyses were performed using a Prominence-1 LC-2030C 3D HPLC system operated via LabSolution software. The system was equipped with two analytical columns: a C18 Hypersil GOLD™ column (100 mm × 4.6 mm, 5 µm particle size; Thermo Scientific, Waltham, MA, USA) and a Chiralpak^®^ HSA column (100 × 4 mm, 5 µm; Daicel Chiral Technologies, West Chester, PA, USA). Guard columns packed with identical stationary phases were used to protect both analytical columns. The aqueous mobile phase (A) consisted of 50 mM ammonium acetate prepared with ultrapure water obtained from a Milli-Q purification system (resistivity 18.2 MΩ) and adjusted to physiological pH 7.4 using concentrated ammonia solution. For lipophilicity measurements, chromatographic separation on the C18 column was carried out at 40 °C with a flow rate of 1.5 mL min^−1^. A linear gradient of acetonitrile (phase B) from 2% to 98% was applied over 5.25 min, followed by an isocratic step at maximum organic content for 2.5 min. Plasma protein binding was assessed on the HSA column using isopropanol as mobile phase B. The gradient program increased isopropanol concentration from 0% to 20% over 15 min, followed by an isocratic stage at 20% for 12 min. The system was then re-equilibrated with pure ammonium acetate solution for 5 min. The column temperature was maintained at 30 °C and the flow rate was set to 0.9 mL min^−1^. Calibration of lipophilicity measurements was performed using the Valko reference mixture (Bio-Mimetic Chromatography Ltd., London, UK). Reference compounds employed for HSA calibration included diclofenac and warfarin (Alfa Aesar, Ward Hill, MA, USA); indomethacin, trimethoprim, propranolol, and paracetamol (Sigma-Aldrich, Steinheim, Germany); nicardipine and nizatidine (Cayman Chemical, Ann Arbor, MI, USA); and carbamazepine (Acros Organics, Geel, Belgium). Test compounds were dissolved in dimethyl sulfoxide to obtain stock solutions at a concentration of 200 µg mL^−1^. UV detection was performed within the wavelength range of 190–300 nm. The injection volume was 5 µL, and all measurements were conducted in duplicate. Retention time reproducibility was within 2%. Chromatographic hydrophobicity index (CHI) values were converted to CHI logD using the following equation [41]:

CHI logD = 0.0525 × CHI − 1.467(1)

For plasma protein binding assessment, retention data from the HSA column were transformed into percentage binding values (%HSA) according to the following relationship [38]:

%HSA = (101 × 10^(logk_HSA))/(1 + 10^(logk_HSA))(2)

## 3. Results and Discussion

### 3.1. Chemistry

**Scheme 1:**
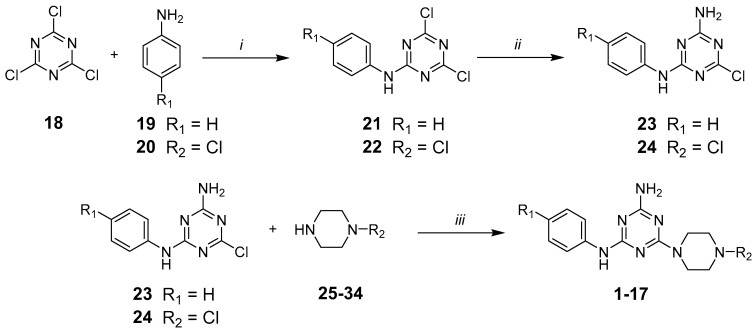
Synthesis pathway of compounds **23**–**24**. *i*—DIPEA, THF, 0–5 °C, 2 hrs; *ii*—NH_3_ (aq, 25%), THF, room temp., 15 h; *iii*—K_2_CO_3_, TBAB, H_2_O, ))), 5 min.

The general procedure for the synthesis of compounds **1**–**17** involved the three-stage sequential *N*-alkylation of cyanuric chloride **18**, performed according to the procedure described in our previous papers [14,42]. In the first step, cyanuric chloride **18** was reacted with aniline **19** or 4-chloroaniline **20** at 0–5 °C in THF in the presence of DIPEA, affording intermediates **21** and **22** in 70–79% of yields, respectively. Subsequently, intermediates **21** and **22** were dissolved in THF, followed by the addition of aqueous ammonia solution. The reaction mixtures were stirred at room temperature overnight, leading to the formation of intermediates **23** and **24** in 83–90% yields, respectively, as brown solids (Scheme 1).

The final step of synthesis of set I and II compounds was performed according to a previously developed sonochemistry protocol. Intermediates **23** and **24** were reacted with commercially available piperazine derivatives (**25**–**33** for set I; **25**–**30** and **33**–**34** for set II). The reactions were carried out in water medium in the presence of potassium carbonate as a base and TBAB as phase-transfer catalyst over 5 min in total (Scheme 1). Final compounds **1**–**17** were obtained in 67–92% yields after purification using chromatographic methods. The structures of the final compounds, along with their corresponding yields, are presented in Table 1.

### 3.2. Antiproliferative Activity

The antiproliferative activities of the obtained compounds against the human breast cancer cell lines MDA-MB-468, MDA-MB-231 and Hs578T, as well as the non-tumorigenic epithelial cell line MCF10A, were evaluated using the MTT assay. Compounds that did not exhibit antiproliferative properties at 100 µM were not subjected to further analysis. As presented in Table 2, majority of compounds demonstrated antiproliferative activity toward the cancer cell lines, particularly in the MDA-MB-231 cell line, which is known to display low sensitivity to cisplatin under short exposure conditions. Although reduced selectivity toward the non-tumorigenic MCF10A cell line was observed, IC_50_ values generally remained higher than those obtained for TNBC cell lines, indicating a moderate but acceptable degree of cancer cell preference at this stage of development.

Compounds **1**, **6**, **8**, **10** and **16** did not exhibit antiproliferative activity against any of the tested cell lines at levels sufficient to determine IC_50_ values within applied incubation time and concentration range. Among all tested 1,3,5-triazine derivatives, only compounds **2** and **7** did not display toxicity toward the non-tumorigenic MCF10A cell line. In both sets, derivatives lacking the aryl substituent (compounds **2** and **8**) showed no cytotoxic activity, suggesting that the aryl fragment is essential for the antiproliferative effect. Comparison of compounds from set I (aniline derivatives) with their analogues from set II (4-chloroaniline derivatives) showed that the effect of halogenation varied depending on the cell line: for MDA-MB-468 and MCF10A, no consistent advantage for either set was observed, while for MDA-MB-231 and Hs578T, the 4-chloroaniline derivatives from set II generally exhibited higher activity. Additionally, evaluation of the activity of compounds **2** and **8** showed that incorporating a methylene linker between the piperazine and phenyl ring (as in compound **8**) dramatically decreases activity.

The introduction of halogen atoms on the phenyl ring of the phenylpiperazine moiety enhanced cytotoxic activity within both sets. In set I, the 2- and 3-chloro-substituted derivatives (compounds **3** and **4**) were the most active, while the 4-chloro-substituted derivative (compound **5**) exhibited slightly lower potency, although still higher than the unsubstituted analogue (compound **2**). The 2-methoxy-substituted derivative **7** showed very low toxicity toward non-tumorigenic MCF10A cell line. Its activity toward cancer cell lines, although lower than that of the halogenated analogues **3**–**5**, remained higher than in the non-substituted compound **2**. Another methoxylated analogue, compound **6**, did not demonstrate significant activity toward any of the tested cell lines.

A similar trend was observed in set II. The 3- and 4-chloro-substituted derivatives (compounds **13** and **14**) showed slightly higher activity than 2-chloro-substituted compound **12**, and all three halogenated derivatives outperformed the unsubstituted compound **11** across the MDA-MB-468, MDA-MB-231, and Hs578T cell lines, while also displaying higher toxicity toward the MCF10A line. The 4-methoxy-substituted compound **15** demonstrated the best activity against the MDA-MB-468 line and the lowest toxicity toward MCF10A among set II derivatives containing the phenylpiperazine moiety (compounds **11**–**16**), while its activity toward other cell lines was lower or comparable to the remaining analogues. In contrast, the 3-methoxy derivative **16** was inactive.

Across both sets, the strongest antiproliferative activity against breast cancer cell lines was observed for compounds **9** and **17**, both containing the 3-(1-piperazinyl)-1,2-benzisothiazole moiety. Since these derivatives also showed the highest toxicity toward the non-tumorigenic MCF10A cel line (IC_50_ = 31.97 μM for **9**, IC_50_ = 39.25 μM for **17**), they were selected for further toxicity evaluation.

The calculation of the selectivity index (SI) for the compounds revealed generally low values across all three cancer cell lines. For most derivatives, including the highly active compounds **9** and **17**, the SI was close to or below 1, or could not be determined, indicating limited selectivity toward cancer cells over the non-tumorigenic MCF10A line. Compounds **12**–**14** exhibited slightly higher SI values for some cell lines; however, these values remain insufficient.

In particular, comparison of substitution patterns within both derivative sets indicates that the presence of an aromatic moiety is crucial for maintaining antiproliferative activity, most likely due to enhanced hydrophobic interactions with biological targets. Furthermore, the consistent activity improvement observed for halogen-substituted analogues suggests that electronic effects and increased lipophilicity play an important role in modulating cellular potency, providing a rational basis for further structural refinement. These findings underscore the need for structural optimization to enhance cancer-specific cytotoxicity while minimizing toxicity to normal cells.

### 3.3. In Vivo Toxicity

**Figure 2:**
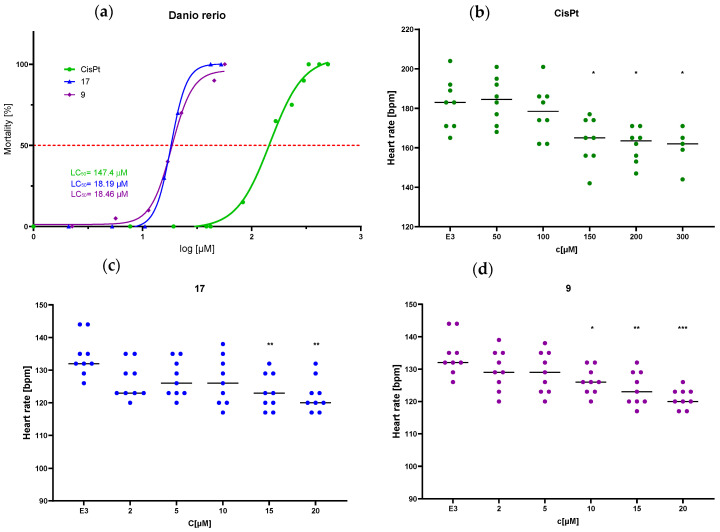
(**a**) Effect of compounds **9** and **7** on *Danio rerio* mortality (n = 9). Cardiotoxicity of (**b**) cisplatin, (**c**) compound **17**, and (**d**) compound **9** on *Danio rerio* model compared with negative control DMSO 1% in growth medium (E3). The statistical significance (GraphPad Prism 8.0.1) was evaluated by a one-way ANOVA, followed by Tukey’s post hoc test (* *p* < 0.05; ** *p* < 0.01; *** *p* < 0.001).

Compounds **9** and **17,** which exhibited the highest antiproliferative activity in the in vitro assay, were evaluated for toxicity using the *Danio rerio* experimental model. The test, conducted according to the OECD protocol with 96 h incubation period [43], allowed for assessment of general toxicity and cardiotoxicity of both compounds (Figure 2).

The general toxicity test demonstrated that the LC_50_ values (Lethal Concentration 50%) for compounds **9** and **17** were 18.46 μM and 18.19 μM, respectively, whereas the LC_50_ of the reference chemotherapeutic agent cisplatin (CisPt) was 147.4 μM under the applied experimental conditions. These data indicate that compounds **9** and **17** induced lethality at lower micromolar concentrations than cisplatin in the zebrafish embryo assay. However, cisplatin is known to exhibit species- and developmental stage-dependent toxicity profiles and therefore should be considered here primarily as an internal reference compound rather than a direct translational comparator. Furthermore, LC_50_ values derived from in vivo lethality endpoints should not be directly compared with IC_50_ values from short-term in vitro antiproliferative assays, as these parameters reflect distinct biological processes and experimental contexts. Therefore, in future studies, a more informative assessment of safety will rely on therapeutic window estimation, defined as the ratio between toxic and effective concentrations under exposure-aligned conditions.

In the cardiotoxicity assay, heart rate tended to decrease with increasing compound concentration; however, up to approximately 10 μM, the observed changes were not statistically significant. This suggests that the general toxicity observed at higher concentrations is unlikely to be primarily driven by an acute effect on cardiac function.

### 3.4. Biomimetic Chromatography—ADME Profiling

Integrating ADME screening with activity studies is essential during the early phases of drug development, as suboptimal pharmacokinetic properties often result in failures during the more costly subsequent stages [37,44,45,46]. At GlaxoSmithKline, Valko and his team developed a biomimetic chromatography platform to assess critical properties in early ADME screening such as lipophilicity and plasma protein binding (PPB). This was achieved through a straightforward chromatographic gradient elution experiment, wherein the retention of analytes was compared with that of model substances. In Table 3, experimentally determined CHI log*D*, and %HSA have been collected.

In general, the molecules under investigation exhibit relatively high lipophilicity, with most analogues possessing CHI logD_7.4_ values in the range of approximately 3–4, a region typically associated with good passive membrane permeability and an overall drug-like character. However, there are two notable exceptions: compounds **2** and **10**, which have significantly lower logD values of 0.84 and 1.84, respectively.

PPB was assessed using chromatographic columns coated with human serum albumin (HSA). HSA is the most abundant protein in human plasma and possesses well-characterized binding sites for xenobiotics. High affinity for plasma proteins can give rise to several safety and efficacy concerns, including reduced clearance, restricted brain penetration and decreased pharmacological response, as only the unbound fraction of a drug is able to interact with its pharmacological targets [47]. In contrast, very weak protein binding may shorten the duration of action and promote rapid elimination [48]. The molecules under investigation demonstrated generally high affinity for HSA. For eight analogues, the extent of binding exceeded that of diclofenac, which constituted the upper limit of the calibration set and corresponds to a quantitative threshold of ≥99.8% HSA binding. Such high protein binding (>99.8%) may represent a potential liability, as it can markedly reduce the free drug fraction and potentially limit tissue penetration. As expected, affinity to HSA correlated strongly with lipophilicity (R^2^ = 0.754). This is because increased lipo-philicity typically promotes stronger HSA binding. However, this relationship was not purely deterministic, indicating that additional molecular features also contribute to PPB. This finding underscores the necessity of assessing PPB experimentally, rather than inferring it solely from lipophilicity, since compounds with otherwise favorable lipophilicity may still display an unacceptable protein-binding profile. From the perspective of further development, particular attention should be focused on compounds **9**, **14** and **17**, which combine the most favorable anticancer activity profiles. Among these, compound **17** appears especially promising, exhibiting a CHI logD_7.4_ of 4.18 and a well-balanced affinity for HSA (92.53%), ensuring a reasonable unbound fraction while maintaining adequate systemic exposure in early ADME assessment. This observation aligns with the broader principle that physico-chemical properties critically influence biological fate [49].

## 4. Conclusions

In this study, a library of 17 novel 1,3,5-triazine derivatives was synthesized with the aim of identifying new small-molecule candidates for the treatment of triple-negative breast cancer (TNBC). An ultrasound-assisted synthetic protocol was implemented, enabling an efficient and more sustainable preparation of the target compounds. The antiproliferative activity of the synthesized derivatives was evaluated in vitro against TNBC cell lines, namely MDA-MB-468, MDA-MB-231 and Hs578T, using the MTT assay. Among the tested compounds, 12 of them exhibited antiproliferative activity against at least two of the cancer cell lines. However, as most compounds also displayed cytotoxicity toward the non-tumorigenic MCF10A cell line, further evaluation of their safety profiles was required.

Compounds **9** and **17**, which showed the most promising in vitro activity against TNBC cell lines, were selected for in vivo assessment using the *Danio rerio* model. General toxicity studies revealed relatively low LC_50_ values of 18.46 μM for compound **9** and 18.19 μM for compound **17**; however, the cardiotoxicity assay showed no significant decrease in heart rate in *Danio rerio* larvae at the tested concentrations, suggesting no adverse effects on cardiac function. It is worth noting that the relatively low LC_50_ values observed in the *Danio rerio* model should not be directly compared with the IC_50_ values obtained in antiproliferative assays, as these experiments were conducted under different exposure conditions. Specifically, in vivo toxicity was assessed over a prolonged 72–96 h exposure period in accordance with OECD guidelines, whereas antiproliferative activity was evaluated after 24 h of compound treatment. Therefore, these parameters reflect distinct biological endpoints and experimental timeframes and should be interpreted independently.

All synthesized compounds were further evaluated for their pharmacokinetic properties, including the chromatographic hydrophobicity index at physiological pH (CHI logD_7.4_) and binding to human serum albumin (%HSA), enabling early ADME profiling. The most favorable balance between antiproliferative activity and pharmacokinetic parameters was observed for compounds **9**, **14**, and **17**. Notably, compound **17** appears to be the most promising candidate, exhibiting a CHI logD_7.4_ value of 4.18 and optimal HSA binding (92.53%).

Further enhancement of biological activity may be achieved through rational structural modification, such as replacing the –NH_2_ or aniline group with a morpholine moiety. This concept is inspired by clinically investigated 1,3,5-triazine derivatives (ZSTK474, gedatolisib, bimiralisib), which contain morpholine fragments and function as pan-PI3K or dual PI3K/mTOR inhibitors [11]. Such modification could facilitate a transition from an activity-based to a more target-oriented design strategy. Subsequent development should also include the assessment of metabolic stability and deeper mechanistic studies, particularly the evaluation of PI3K/mTOR pathway modulation. Validation in advanced TNBC models, including 3D cultures and patient-derived organoids, would further strengthen translational relevance. Additionally, formulation strategies aimed at improving the therapeutic index may support the optimization of safety and efficacy profiles.

Overall, compounds **9** and **17** showed the highest antiproliferative activity against TNBC cell lines within the synthesized library of compounds, while maintaining satisfying pharmacokinetic properties and an absence of cardiotoxic effects in vivo. However, the elevated general toxicity observed in the *Danio rerio* model, together with the presence of a limited selectivity index due to toxicity toward the non-tumorigenic epithelial cell line MCF10A, indicates that further toxicological studies in more human-relevant in vivo systems and structural optimization are necessary to improve both the efficacy and safety profiles of the compounds. These findings support continued investigation of these compounds in the context of TNBC research.

## Figures and Tables

**Table 1:** Obtained compound library with reaction yields.

Set No.	Cmpd No.	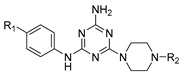		Yield [%]
		R_1_	R_2_	
I	1	H	CH_3_	83
	2	H	Ph	87
	3	H	o-Cl-Ph	69
	4	H	m-Cl-Ph	72
	5	H	p-Cl-Ph	68
	6	H	p-OMe-Ph	90
	7	H	o-OMe-Ph	82
	8	H	Bn	89
	9	H	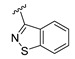	67
II	10	Cl	CH_3_	85
	11	Cl	Ph	78
	12	Cl	o-Cl-Ph	70
	13	Cl	m-Cl-Ph	72
	14	Cl	p-Cl-Ph	80
	15	Cl	p-OMe-Ph	78
	16	Cl	m-OMe-Ph	68
	17	Cl	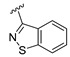	74

**Table 2:** Antiproliferative activity of the synthesized compounds; n.d.—not determined; n.a—not available.

Cmpd No.	MDA-MB-468	MDA-MB-231	Hs578T	MCF10A	SI_MDA-MB-468_	SI_MDA-MB-231_	SI_Hs578T_
	IC_50_ ± SD [µM]	IC_50_ ± SD [µM]	IC_50_ ± SD [µM]	IC_50_ ± SD [µM]			
1	n.d.	n.d.	n.d.	n.d.	n.a	n.a	n.a
2	80.45 ± 11.5	82.80 ± 9.3	n.d.	n.d.	n.a	n.a	n.a
3	60.45 ± 7.0	60.34 ± 6.6	71.91 ± 7.5	63.76 ± 3.9	1.05	1.06	0.89
4	58.99 ± 5.2	63.44 ± 5.1	72.76 ± 8.3	64.12 ± 4.3	1.09	1.01	0.88
5	67.68 ± 23.7	75.79 ± 1.6	n.d.	59.51 ± 22.2	0.88	0.79	n.a
6	n.d.	n.d.	n.d.	n.d.	n.a	n.a	n.a
7	69.53 ± 9.5	83.00 ± 8.6	79.19 ± 13.8	n.d.	n.a	n.a	n.a
8	n.d.	n.d.	n.d.	n.d.	n.a	n.a	n.a
9	36.62 ± 0.9	37.32 ± 1.8	57.26 ± 21.1	31.97 ± 8.5	0.87	0.86	0.56
10	n.d.	n.d.	n.d.	n.d.	n.a	n.a	n.a
11	92.78 ± 4.5	72.88 ± 2.1	64.24 ± 17.9	76.42 ± 2.6	0.82	1.05	1.19
12	61.25 ± 17.6	38.95 ± 7.0	61.83 ± 19.5	68.46 ± 6.6	1.12	1.76	1.11
13	58.97 ± 18.3	36.23 ± 5.6	55.60 ± 14.4	66.03 ± 25.6	1.12	1.82	1.19
14	55.81 ± 9.9	37.50 ± 3.0	62.42 ± 15.2	48.95 ± 12.2	0.88	1.31	0.78
15	47.52 ± 15.8	53.10 ± 9.5	74.62 ± 7.3	76.53 ± 6.2	1.61	1.44	1.03
16	n.d.	n.d.	n.d.	n.d.	n.a	n.a	n.a
17	38.29 ± 7.5	32.86 ± 10.1	34.87 ± 7.7	39.25 ± 13.9	1.03	1.19	1.13
CisPt	6.0 ± 0.5	n.d.	14.1 ± 2.4	n.d.	n.a	n.a	n.a

**Table 3:** Chromatographically determined properties of tested molecules.

Compound No.	CHI log D_7.4_	%HSA
1	n.d.	n.d.
2	0.84	59.76
3	3.92	>99.80
4	3.92	>99.80
5	3.83	>99.80
6	3.00	96.19
7	3.14	96.47
8	3.04	95.42
9	3.63	>99.80
10	1.84	90.17
11	3.83	>99.80
12	4.43	>99.80
13	4.37	>99.80
14	4.31	>99.80
15	n.d.	n.d.
16	n.d.	n.d.
17	4.18	92.53

## Data Availability

Original contributions presented in this study are available within the article. Further inquiries can be directed to the corresponding author.

## Supplementary Materials

The following supporting information can be downloaded at: https://www.mdpi.com/article/10.3390/cimb48030319/s1.
