# Supplementary material for: Ultrasound-Assisted Synthesis and Biological Profiling of 1,3,5-Triazine Derivatives with Antiproliferative Activity in Triple-Negative Breast Cancer
Source: Curr Issues Mol Biol. 2026 Mar 17;48(3):319. doi: 10.3390/cimb48030319 (PMC13025726; doi:10.3390/cimb48030319)

Ultrasound-assisted synthesis and biological profiling of 1,3,5-triazine derivatives with antiproliferative activity in Triple-Negative Breast Cancer

Natalia Bosak [1], Anna Karolina Drabczyk [1], Jolanta Jaśkowska [1], Martyna Stachowicz-Suhs [2,3], Beata Filip-Psurska[4], Anna Boguszewska-Czubara [5], Katarzyna Ewa Greber [6], Krzesimir Ciura [6], and Damian Kułaga [1*]

[1] Department of Organic Chemistry and Technology, Faculty of Chemical Engineering and Technology, Cracow University of Technology, 24 Warszawska Street, 31-155 Cracow, Poland; natalia.bosak54@student.pk.edu.pl (N.B.); anna.drabczyk@pk.edu.pl (A.K.D.); jolanta.jaskowska@pk.edu.pl (J.J.), damian.kulaga@pk.edu.pl (D.K.)

[2] Department of Experimental Oncology, Hirszfeld Institute of Immunology and Experimental Therapy, Polish Academy of Sciences, 12 Weigla Street, 53-114 Wroclaw, Poland

[3] Innate Immunity Research Group, Life Sciences and Biotechnology Center, Łukasiewicz Research Network - PORT Polish Center for Technology Development, 147 Stabłowicka Street, 54-066 Wrocław, Poland; martyna.stachowicz-suhs@port.lukasiewicz.gov.pl

[4] Laboratory of Experimental Anticancer Therapy, Hirszfeld Institute of Immunology and Experimental Therapy, Polish Academy of Sciences, 12 Weigla Street, 53-114 Wroclaw, Poland; beata.filip-psurska@hirszfeld.pl

[5] Department of Medical Chemistry, Medical University of Lublin, 4a Chodźki Street, 20-093 Lublin, Poland; anna.boguszewska-czubara@umlub.pl (A.B.C.)

[6] Department of Physical Chemistry, Faculty of Pharmacy, Medical University of Gdansk, 107 General Jozef Haller Avenue, 80-416 Gdansk, Poland; katarzyna.greber@gumed.edu.pl (K.E.G.); krzesimir.ciura@gumed.edu.pl (K.C.)

* Correspondence: damian.kulaga@pk.edu.pl (D.K.)

Supplementary Material

Supplementary Material contains ^{1}H NMR and ESI spectra only

^{1}H NMR

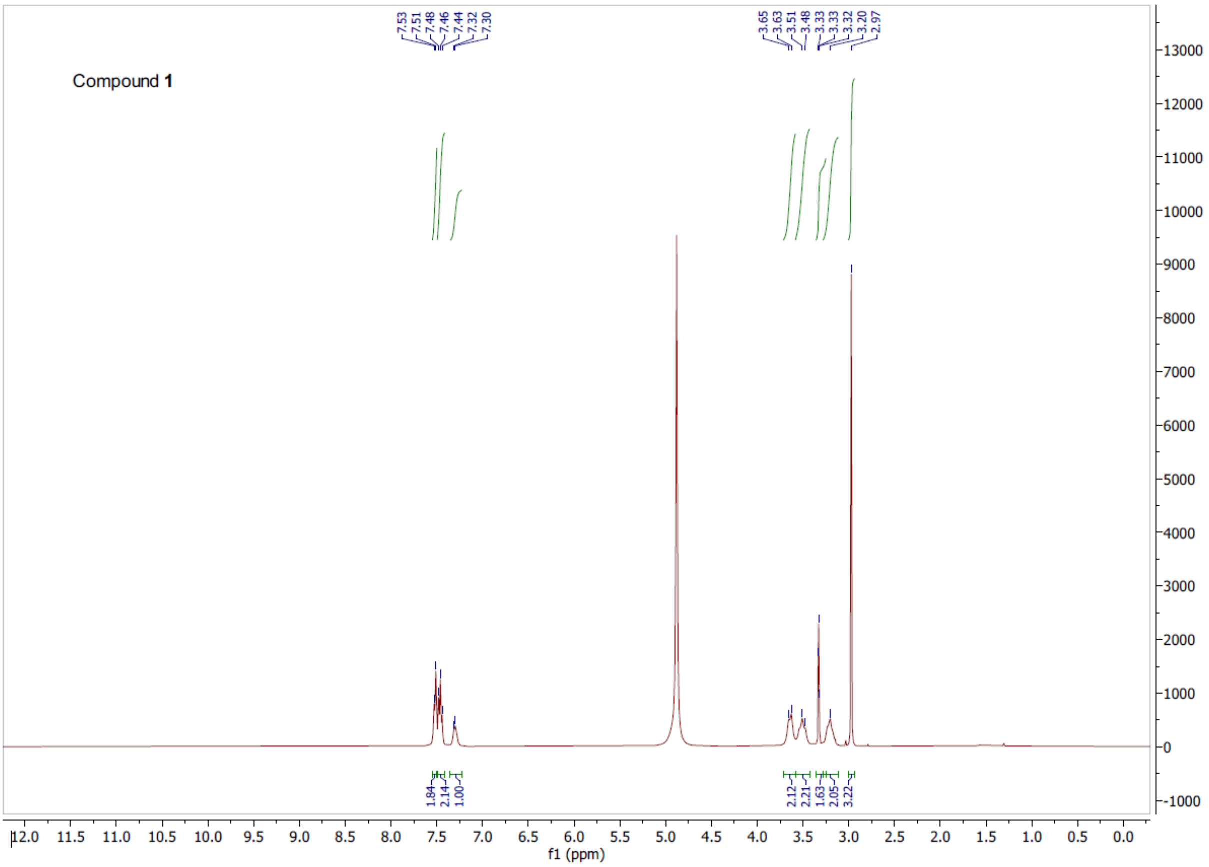

Compound 1
7.53
7.51
7.48
7.46
7.44
7.32
7.30
3.65
3.63
3.51
3.48
3.33
3.33
3.32
3.20
2.97
1.84
2.14
1.00
2.12
2.21
1.63
2.05
3.22
f1 (ppm)

ESI

Formula Predictor Report

Printed at 08.03.2026 13:09:10

Formula Predictor Result	**C14 H19 N7**
Mass	286.177
Error Margin	10 ppm
DBE Range	Not Used
Electron Ions	Both configurations
HC Ratio	Not Used
Nitrogen Rule	Used

#	Score	Pred. (M)	Pred. m/z	Meas. m/z	Diff. (mDa)	Formulae (M)	Ion	Diff. (ppm)	Iso Score	DBE
1	100.00	285.17019	286.17747	286.17700	-0.47	C14 H19 N7	[M+H]+	-1.643	100.00	9.0

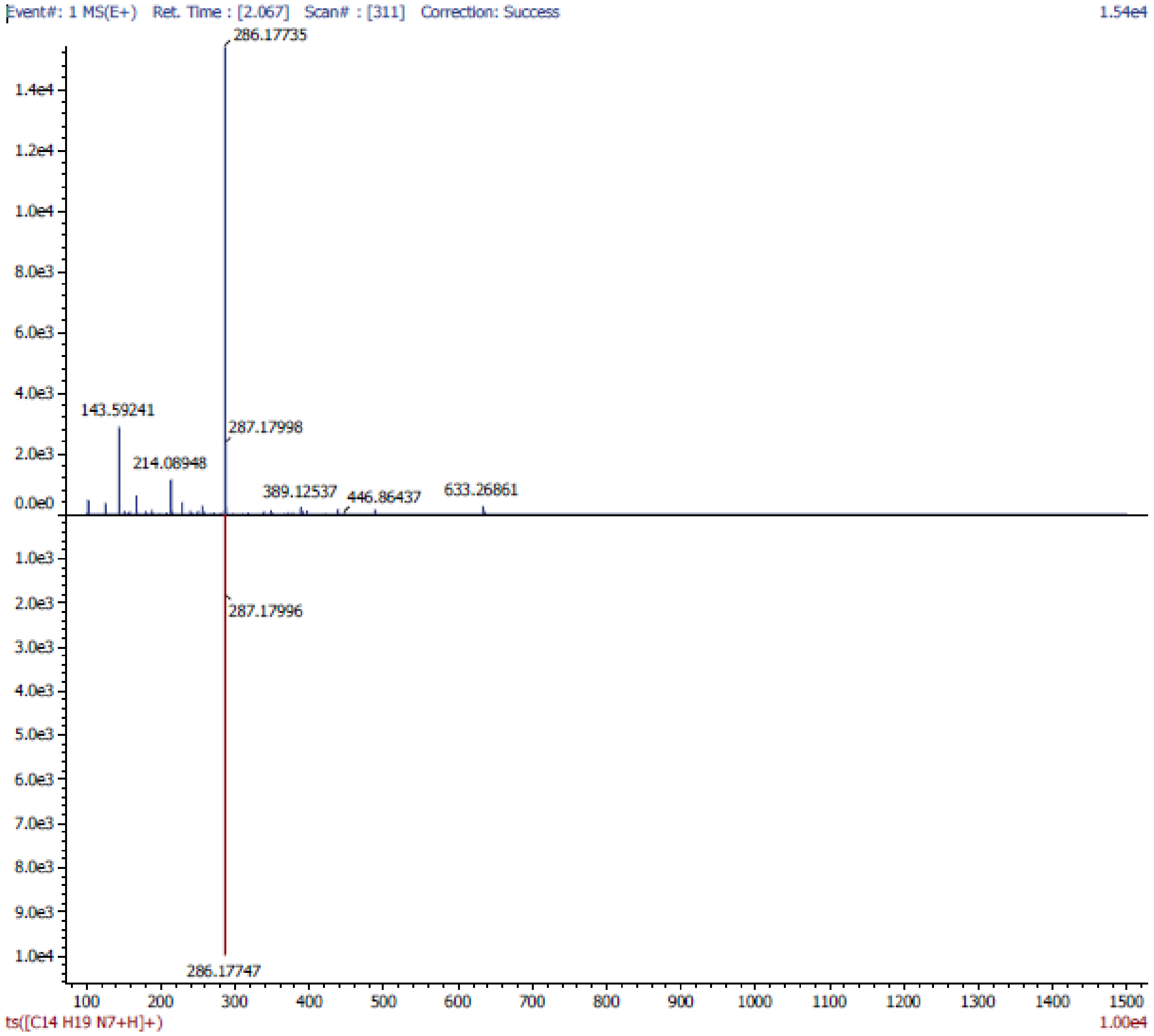

^{1}H NMR

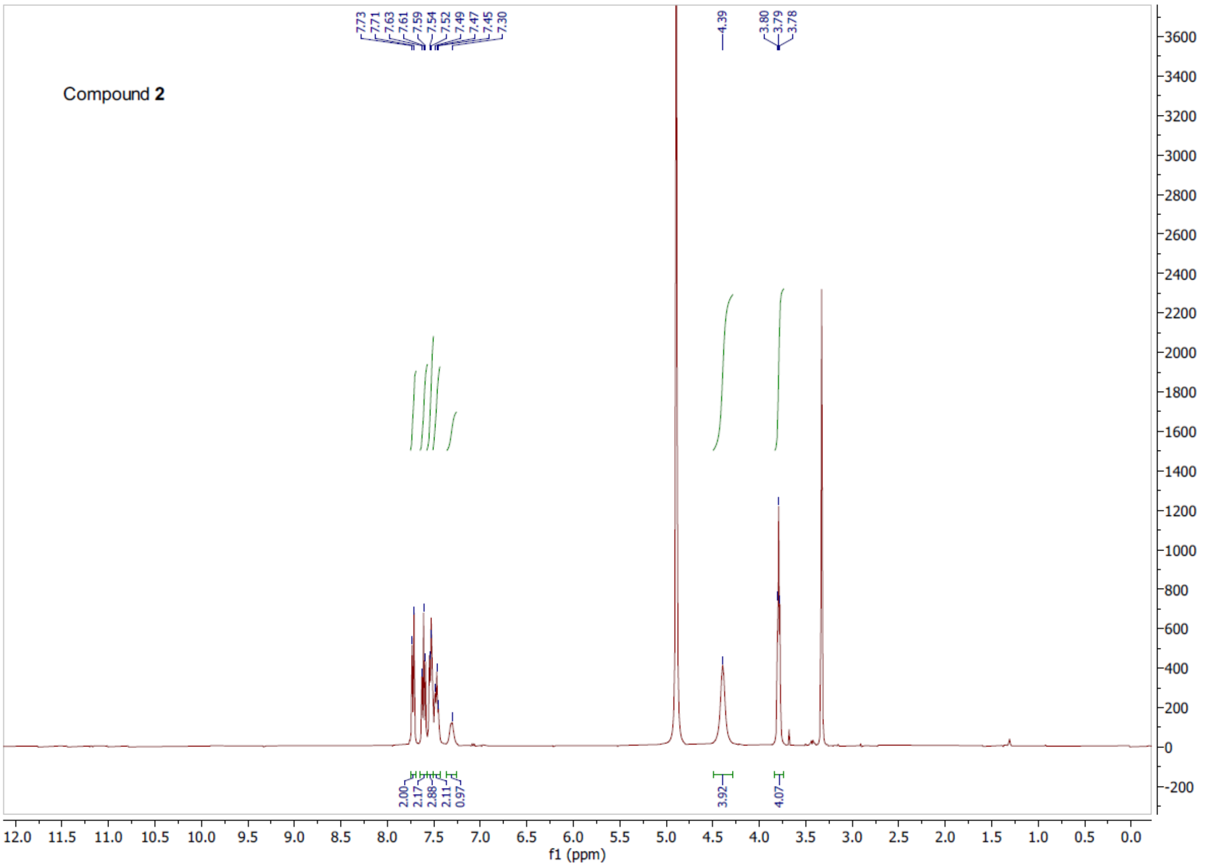

Compound 2
7.73
7.71
7.63
7.61
7.59
7.54
7.52
7.49
7.47
7.45
7.30
4.39
3.80
3.79
3.78
2.00
2.17
2.88
2.11
0.97
3.92
4.07
f1 (ppm)

ESI

Formula Predictor Report

Printed at 04.03.2026 13:00:49

Formula Predictor Result	**C19 H21 N7**
Mass	348.192967203
Error Margin	10 ppm
DBE Range	Not Used
Electron Ions	Both configurations
HC Ratio	Not Used
Nitrogen Rule	Used

#	Score	Pred. (M)	Pred. m/z	Meas. m/z	Diff. (mDa)	Formulae (M)	Ion	Diff. (ppm)	Iso Score	DBE
1	99.98	347.18584	348.19312	348.19297	-0.15	C19 H21 N7	[M+H]+	-0.439	99.97	13.0

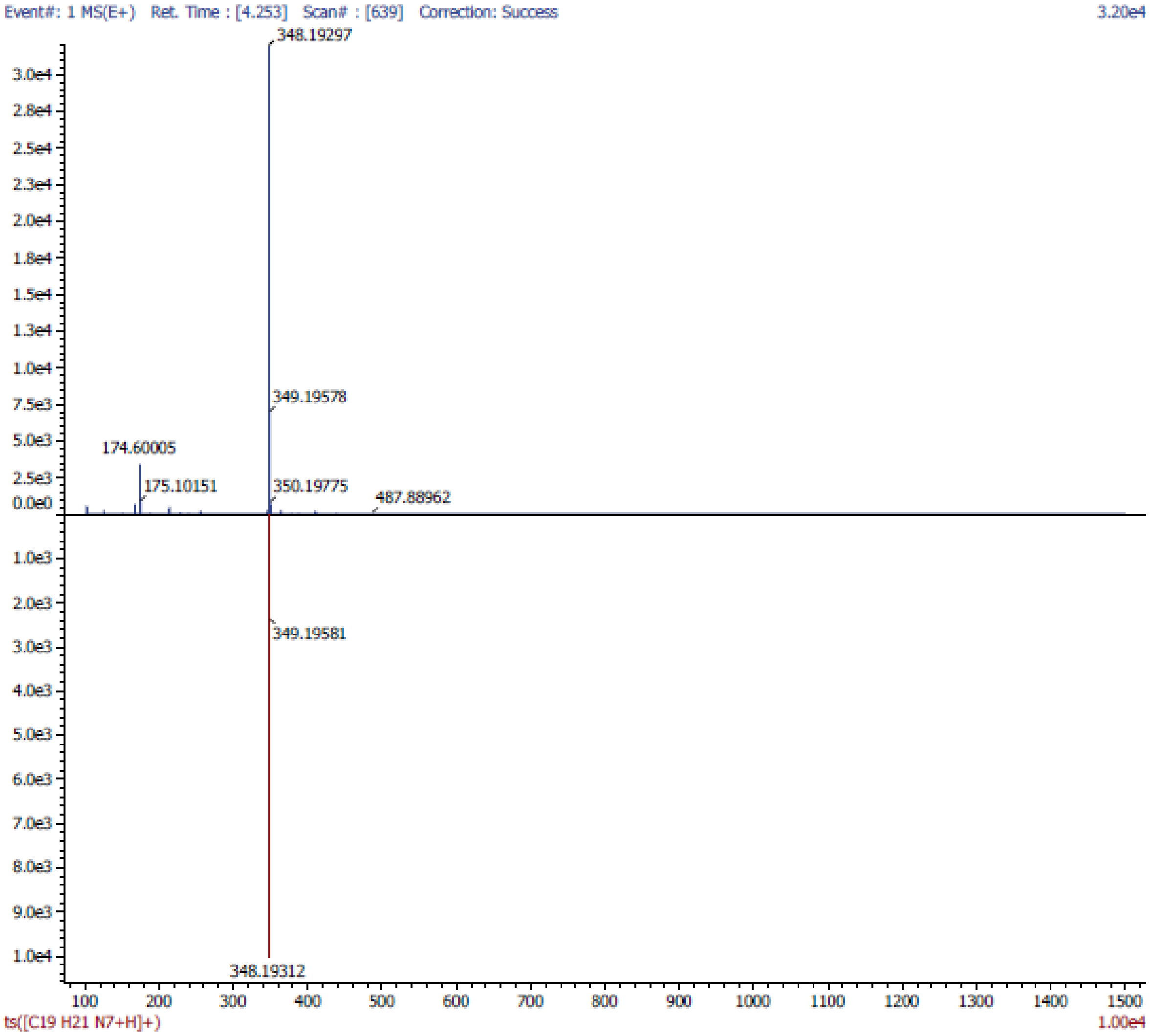

^{1}H NMR

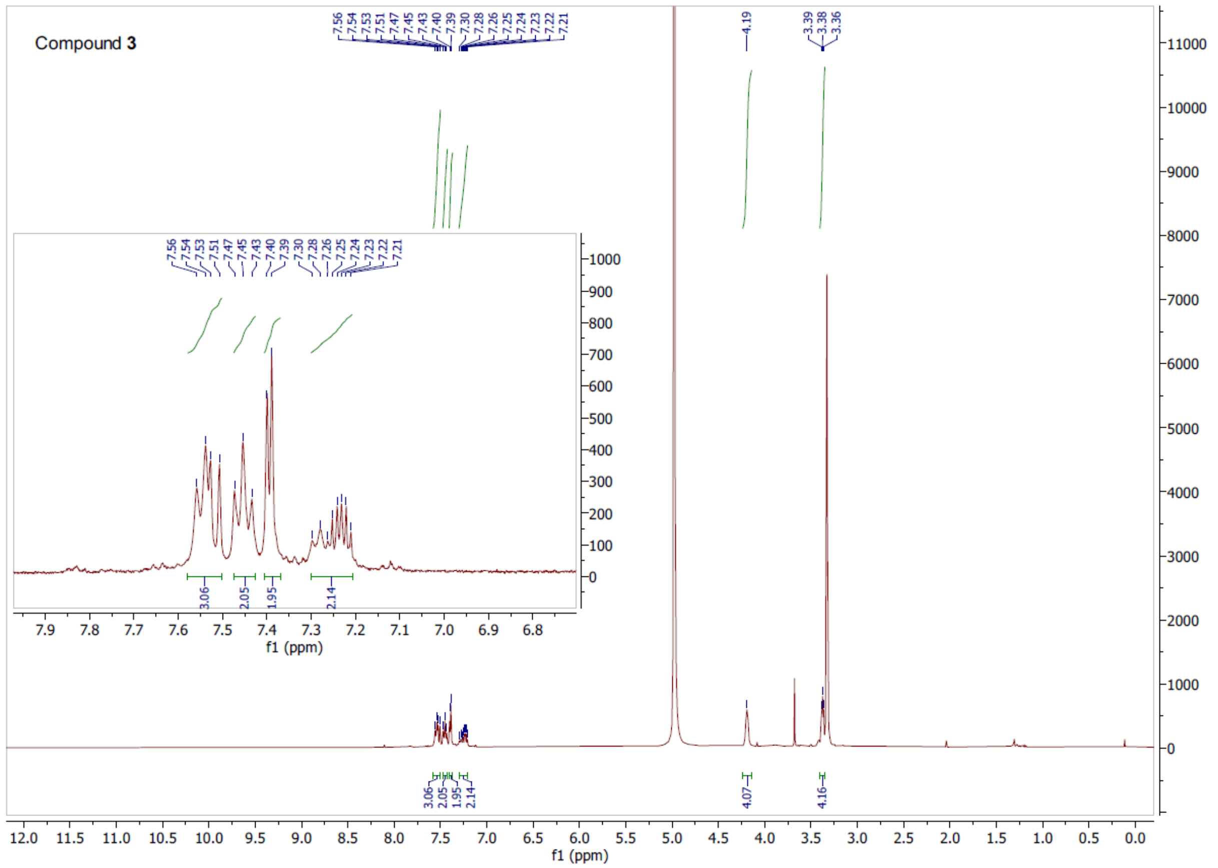

ESI

Formula Predictor Report

Printed at 04.03.2026 13:01:35

Formula Predictor Result	**C19 H20 N7 Cl**
Mass	382.153967234
Error Margin	10 ppm
DBE Range	Not Used
Electron Ions	Both configurations
HC Ratio	Not Used
Nitrogen Rule	Used

#	Score	Pred. (M)	Pred. m/z	Meas. m/z	Diff. (mDa)	Formulae (M)	Ion	Diff. (ppm)	Iso Score	DBE
1	99.14	381.14687	382.15415	382.15397	-0.18	C19 H20 N7 Cl	[M+H]+	-0.473	99.05	13.0

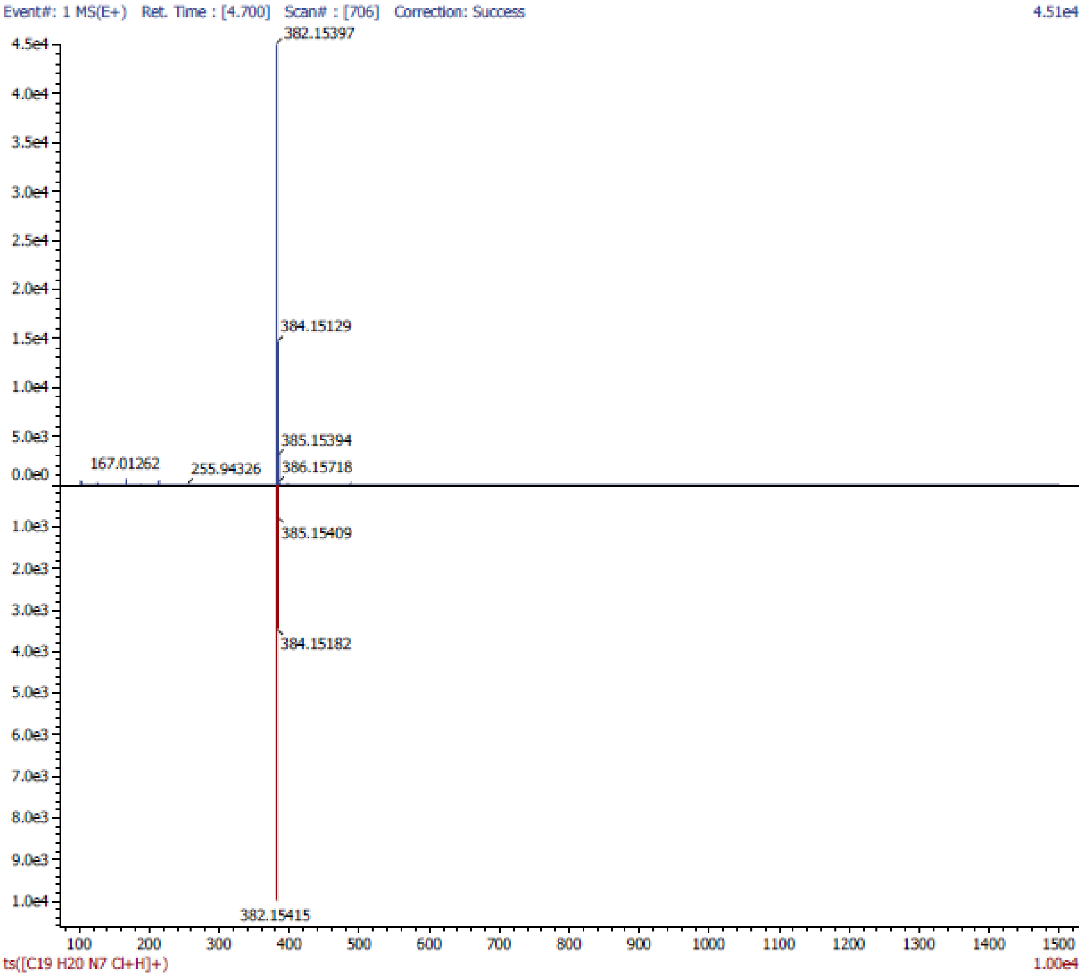

^{1}H NMR

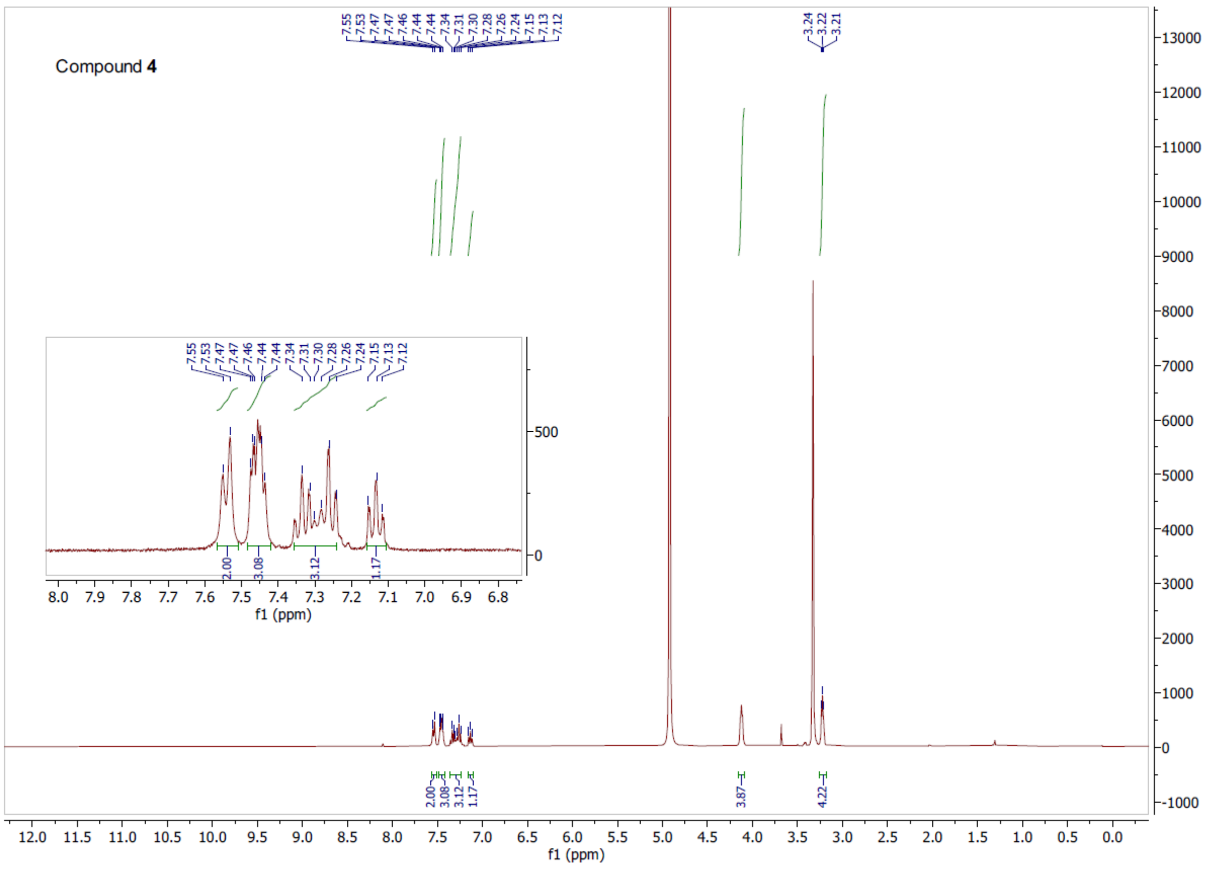

Compound 4
7.55
7.53
7.47
7.47
7.46
7.44
7.44
7.34
7.31
7.30
7.28
7.26
7.24
7.15
7.13
7.12
3.24
3.22
3.21
2.00
3.08
3.12
1.17
3.87
4.22
f1 (ppm)

ESI

Formula Predictor Report

Printed at 04.03.2026 12:59:33

Formula Predictor Result	**C19 H20 N7 Cl**
Mass	382.153904504
Error Margin	10 ppm
DBE Range	Not Used
Electron Ions	Both configurations
HC Ratio	Not Used
Nitrogen Rule	Used

#	Score	Pred. (M)	Pred. m/z	Meas. m/z	Diff. (mDa)	Formulae (M)	Ion	Diff. (ppm)	Iso Score	DBE
1	98.53	381.14687	382.15415	382.15390	-0.24	C19 H20 N7 Cl	[M+H]+	-0.637	98.36	13.0

Event#: 1 MS(E+) Ret. Time : [4.713] Scan# : [708] Correction: Success 2.32e4

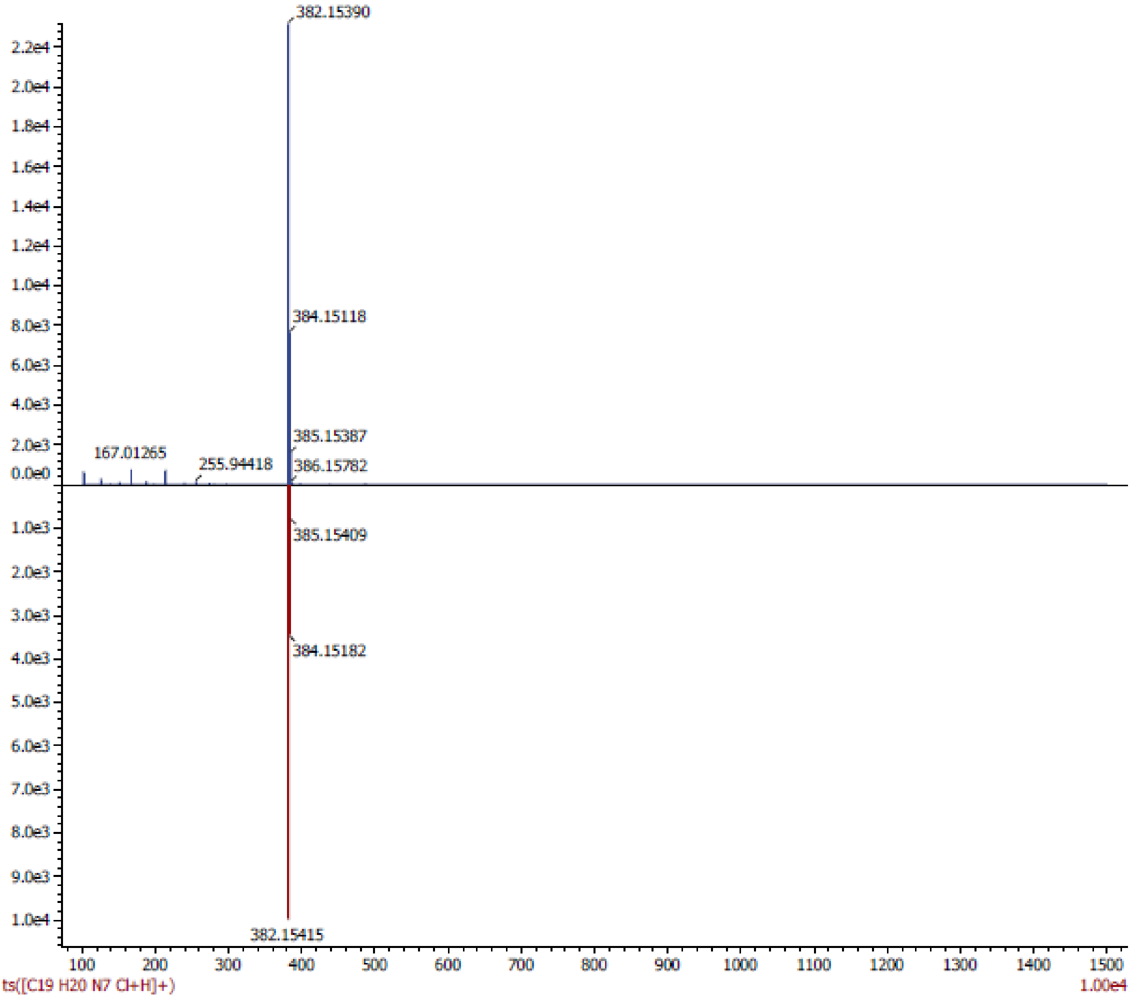

ts([C19 H20 N7 Cl+H]+) 1.00e4

^{1}H NMR

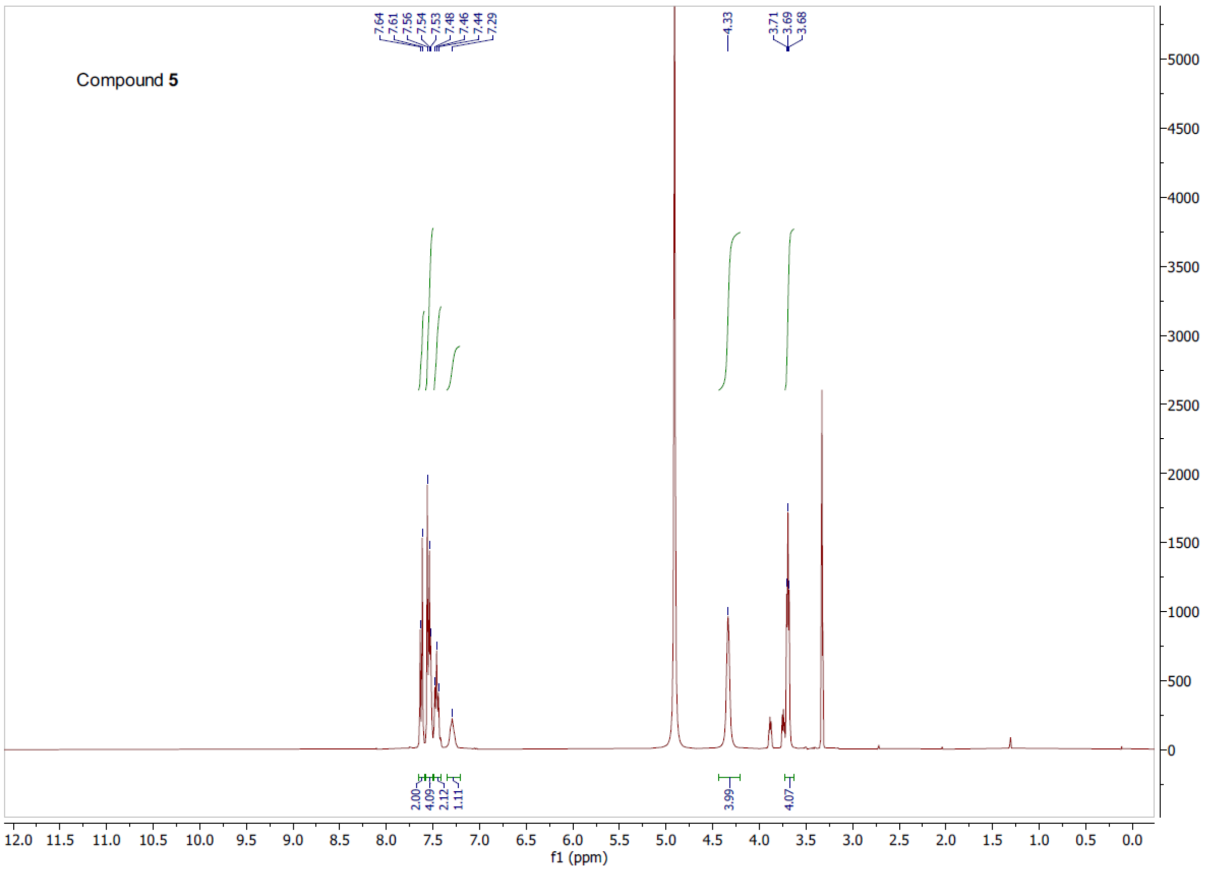
Compound 5
7.64
7.61
7.56
7.54
7.53
7.48
7.46
7.44
7.29
4.33
3.71
3.69
3.68
2.00
4.09
2.12
1.11
3.99
4.07
f1 (ppm)

ESI

Formula Predictor Report

Printed at 04.03.2026 13:13:48

Formula Predictor Result	**C19 H20 N7 Cl**
Mass	382.153904266
Error Margin	10 ppm
DBE Range	Not Used
Electron Ions	Both configurations
HC Ratio	Not Used
Nitrogen Rule	Used

#	Score	Pred. (M)	Pred. m/z	Meas. m/z	Diff. (mDa)	Formulae (M)	Ion	Diff. (ppm)	Iso Score	DBE
1	98.81	381.14687	382.15415	382.15390	-0.24	C19 H20 N7 Cl	[M+H]+	-0.637	98.68	13.0

Event#: 1 MS(E+) Ret. Time : [4.693] Scan# : [705] Correction: Success 3.51e4

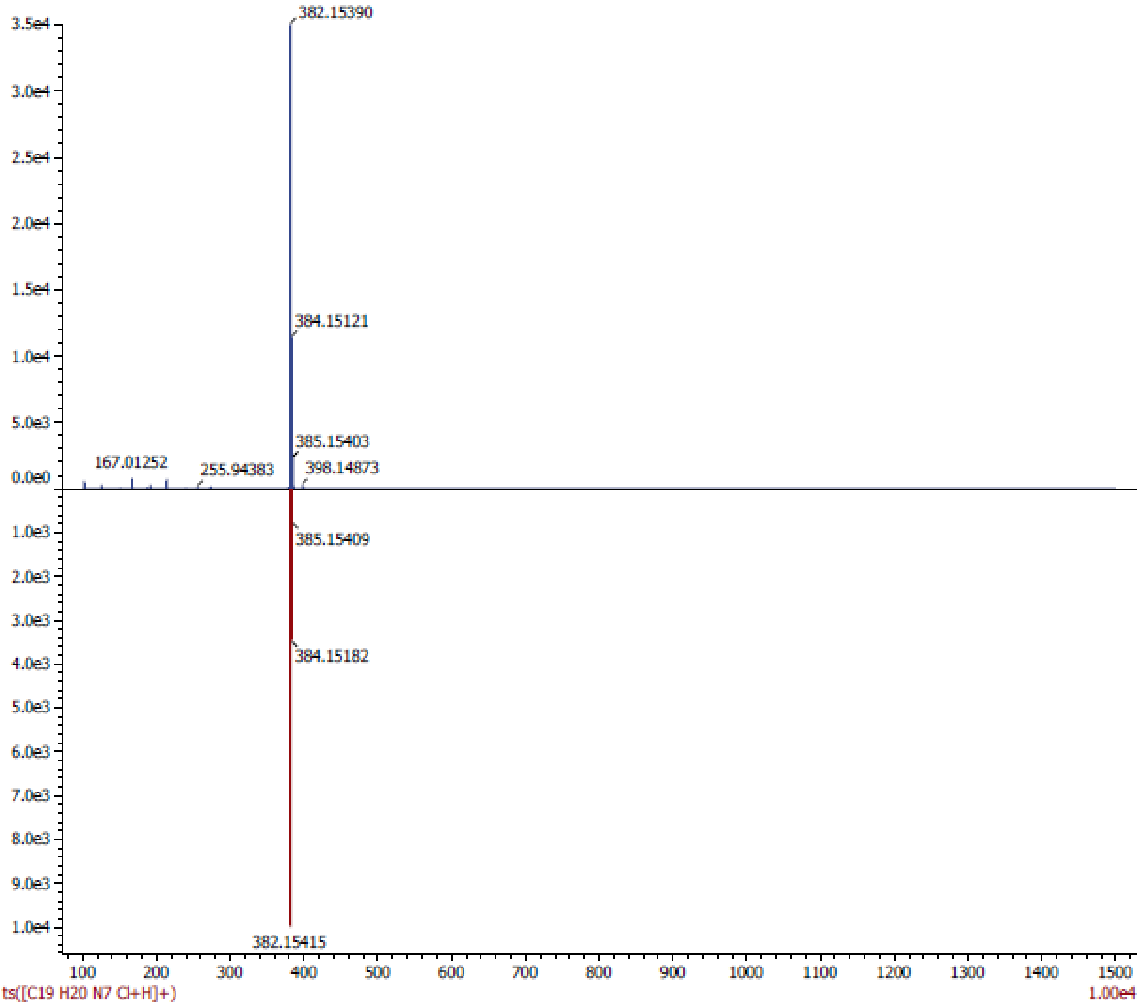

ts([C19 H20 N7 Cl+H]+) 1.00e4

^{1}H NMR

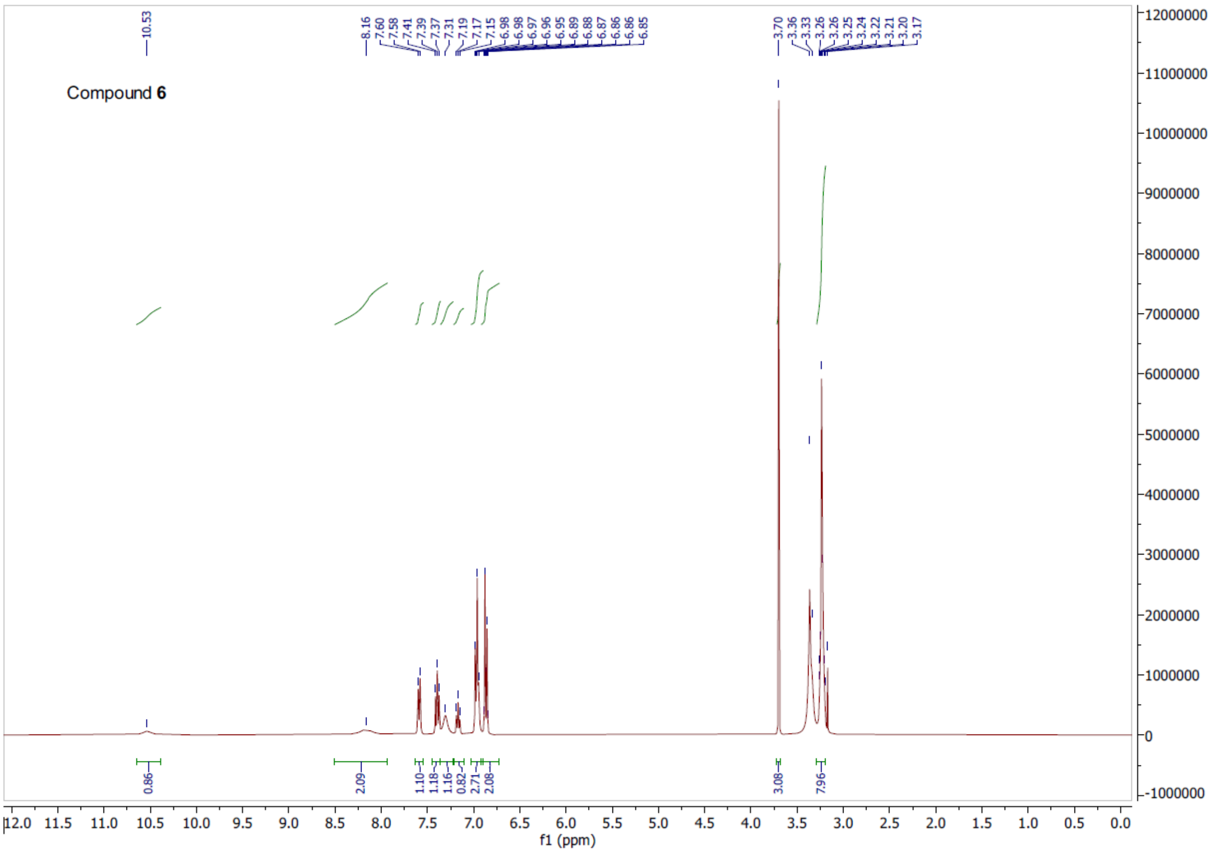

ESI

Formula Predictor Report

Printed at 04.03.2026 12:26:08

Formula Predictor Result	**C20 H23 N7 O**
Mass	378.203
Error Margin	10 ppm
DBE Range	Not Used
Electron Ions	Both configurations
HC Ratio	Not Used
Nitrogen Rule	Used

#	Score	Pred. (M)	Pred. m/z	Meas. m/z	Diff. (mDa)	Formulae (M)	Ion	Diff. (ppm)	Iso Score	DBE
1	98.72	377.19641	378.20368	378.20300	-0.68	C20 H23 N7 O	[M+H]+	-1.811	98.58	13.0

Event#: 1 MS(E+) Ret. Time : [4.107] Scan# : [617] Correction: Success 6.92e3

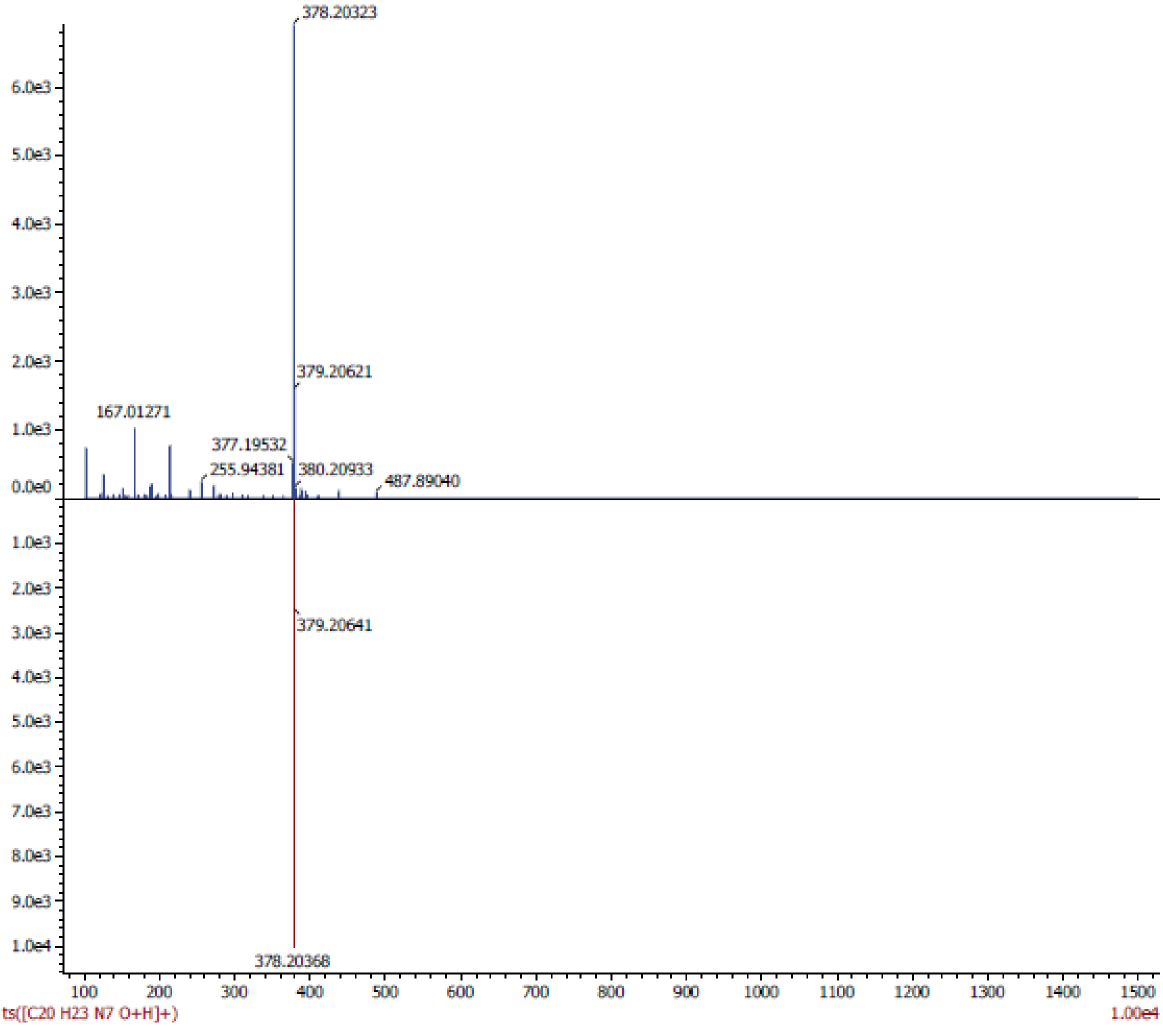

ts([C20 H23 N7 O+H]+) 1.00e4

^{1}H NMR

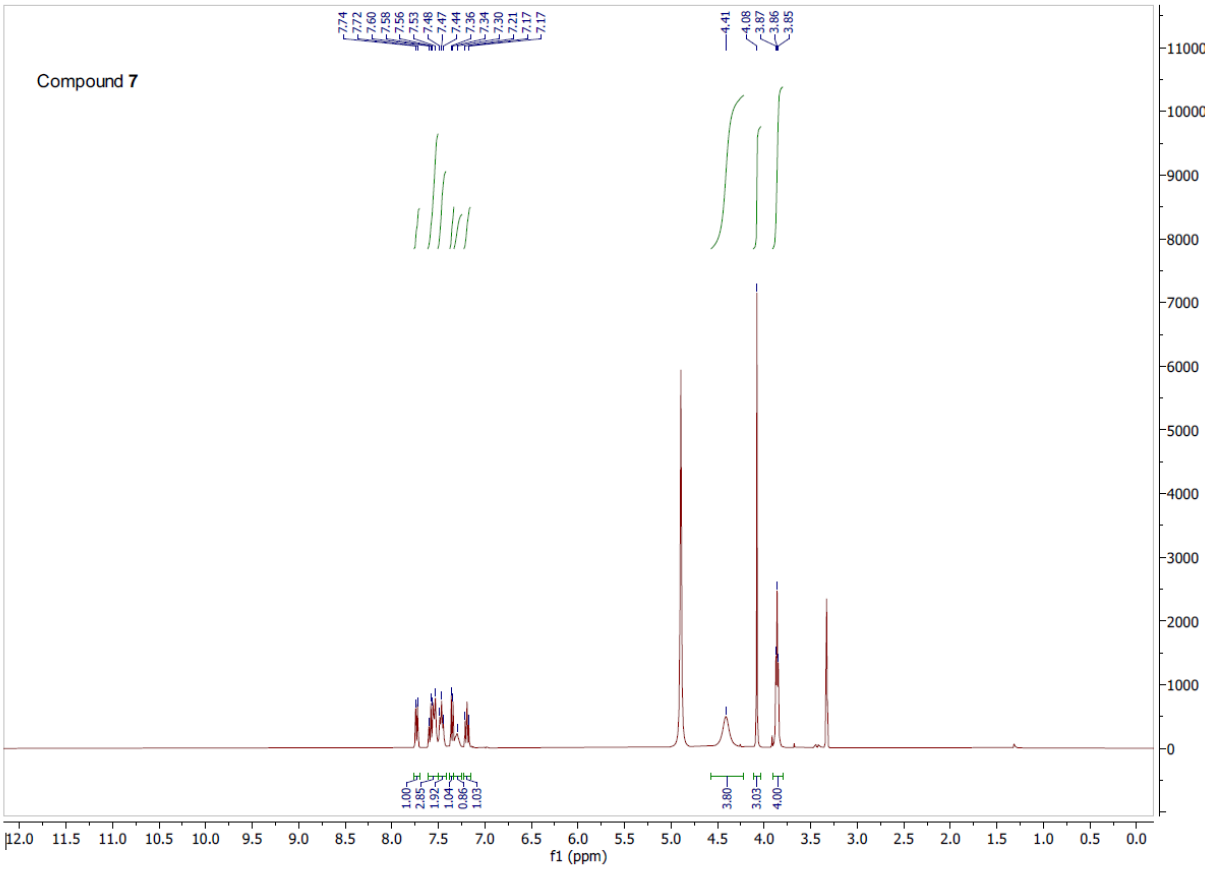

Compound **7**
7.74
7.72
7.60
7.58
7.56
7.53
7.48
7.47
7.44
7.36
7.34
7.30
7.21
7.17
7.17
4.41
4.08
3.87
3.86
3.85
1.00
2.85
1.92
1.04
0.86
1.03
3.80
3.03
4.00
f1 (ppm)

Formula Predictor Report

Printed at 04.03.2026 12:57:11

Formula Predictor Result	**C20 H23 N7 O**
Mass	378.203340345
Error Margin	10 ppm
DBE Range	Not Used
Electron Ions	Both configurations
HC Ratio	Not Used
Nitrogen Rule	Used

#	Score	Pred. (M)	Pred. m/z	Meas. m/z	Diff. (mDa)	Formulae (M)	Ion	Diff. (ppm)	Iso Score	DBE
1	98.99	377.19641	378.20368	378.20334	-0.34	C20 H23 N7 O	[M+H]+	-0.911	98.88	13.0

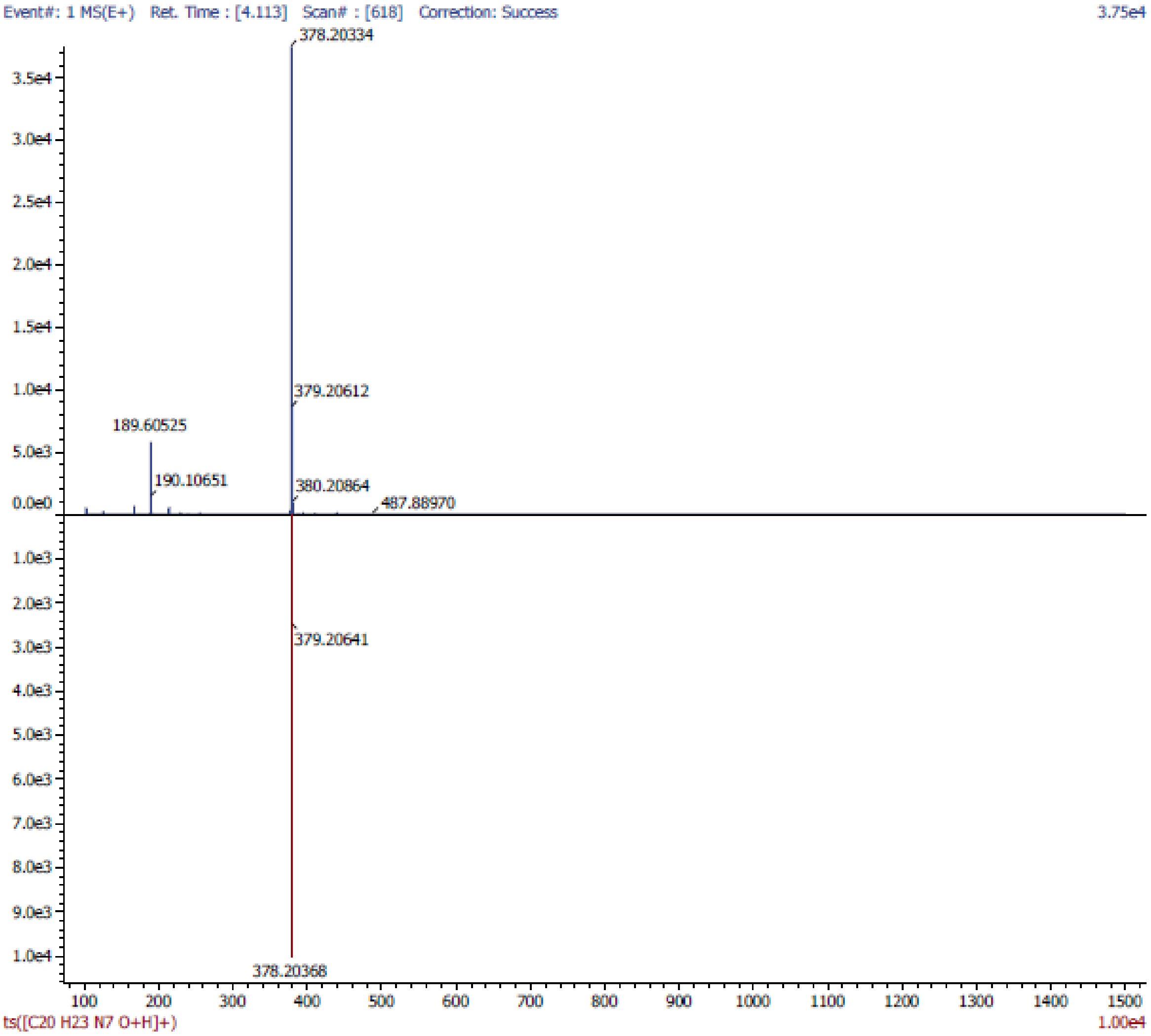

[1] HNMR

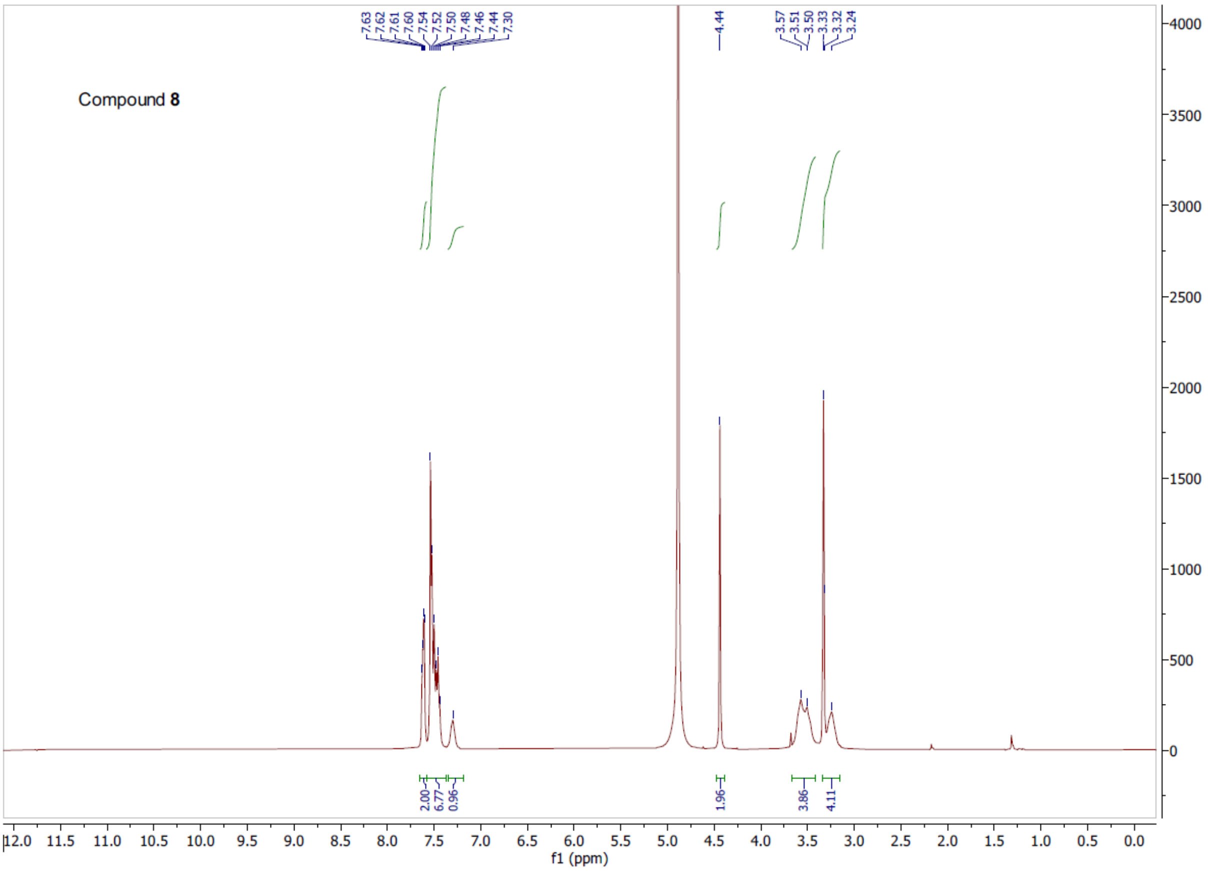
Compound 8
7.63
7.62
7.61
7.60
7.54
7.52
7.50
7.48
7.46
7.44
7.30
4.44
3.57
3.51
3.50
3.33
3.32
3.24
2.00
6.77
0.96
1.96
3.86
4.11
f1 (ppm)

ESI

Formula Predictor Report

Printed at 04.03.2026 13:03:05

Formula Predictor Result	**C20 H23 N7**
Mass	362.208443505
Error Margin	10 ppm
DBE Range	Not Used
Electron Ions	Both configurations
HC Ratio	Not Used
Nitrogen Rule	Used

#	Score	Pred. (M)	Pred. m/z	Meas. m/z	Diff. (mDa)	Formulae (M)	Ion	Diff. (ppm)	Iso Score	DBE
1	99.14	361.20149	362.20877	362.20844	-0.33	C20 H23 N7	[M+H]+	-0.902	99.05	13.0

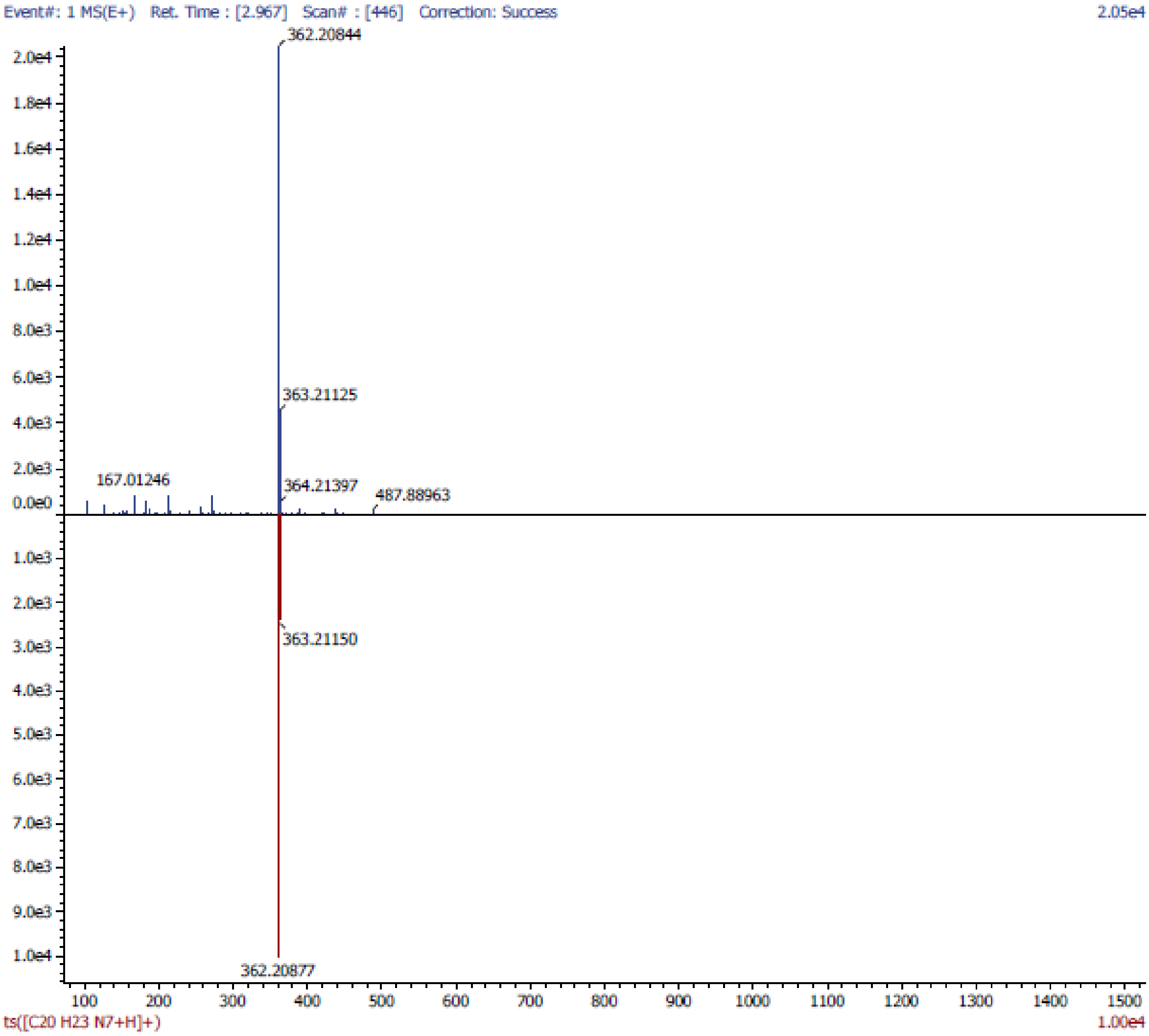

¹H NMR

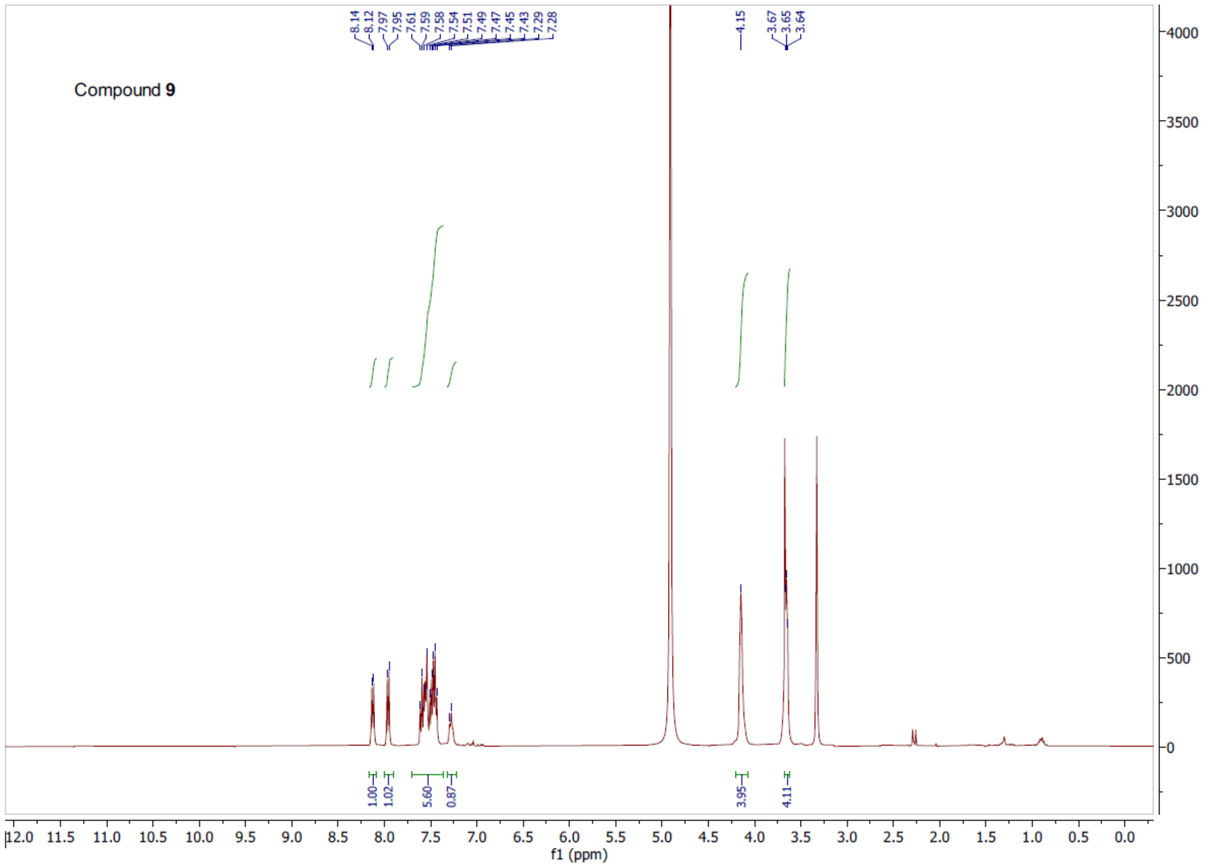

Compound 9
8.14
8.12
7.97
7.95
7.61
7.59
7.58
7.54
7.51
7.49
7.47
7.45
7.43
7.29
7.28
4.15
3.67
3.65
3.64
1.00
1.02
5.60
0.87
3.95
4.11
f1 (ppm)

ESI

Formula Predictor Report

Printed at 04.03.2026 12:52:15

Formula Predictor Result	**C20 H20 N8 S**
Mass	405.1602
Error Margin	100 ppm
DBE Range	Not Used
Electron Ions	Both configurations
HC Ratio	Not Used
Nitrogen Rule	Used

#	Score	Pred. (M)	Pred. m/z	Meas. m/z	Diff. (mDa)	Formulae (M)	Ion	Diff. (ppm)	Iso Score	DBE
1	93.67	404.15316	405.16044	405.16020	-0.24	C20 H20 N8 S	[M+H]+	-0.593	92.96	15.0

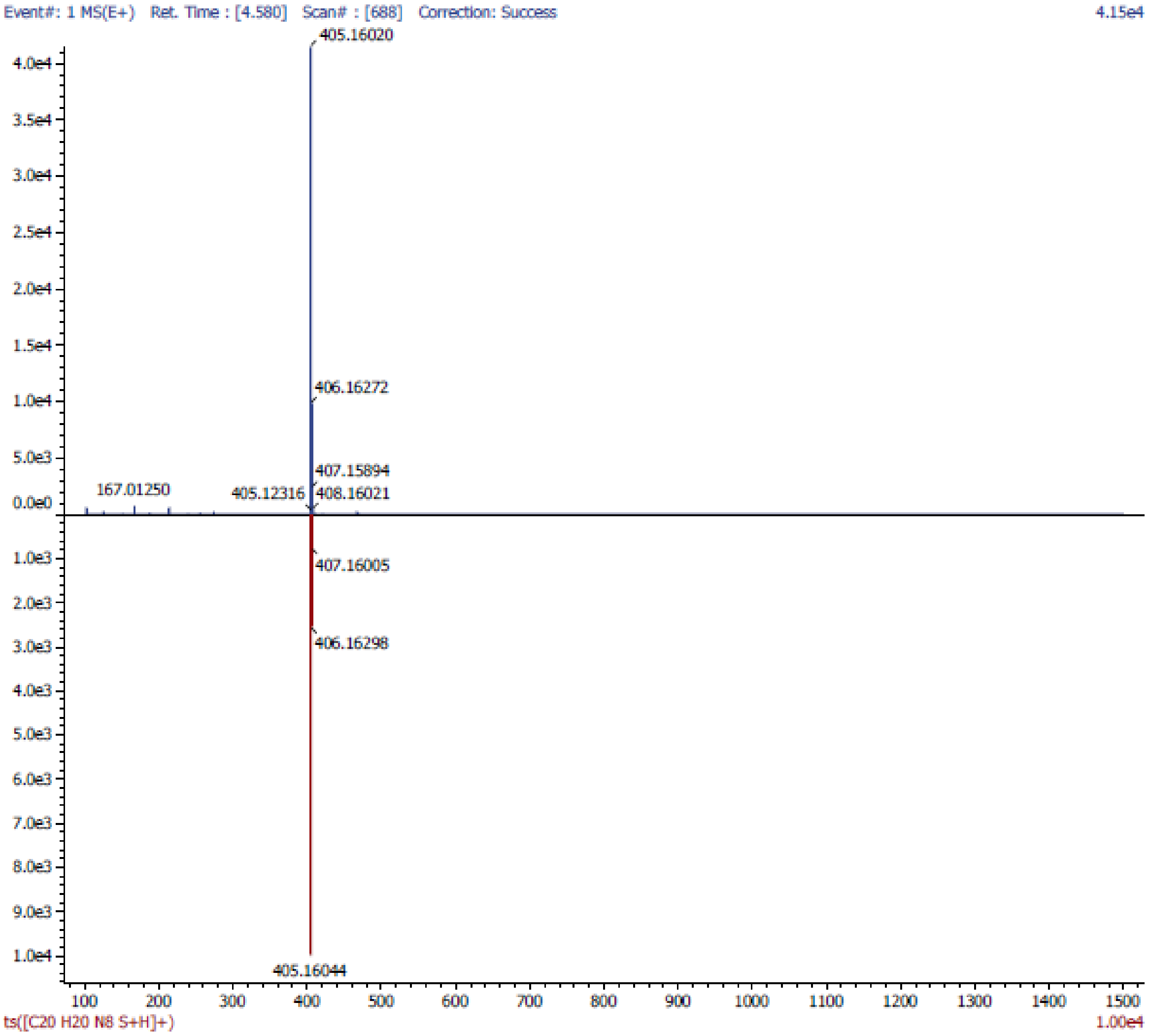

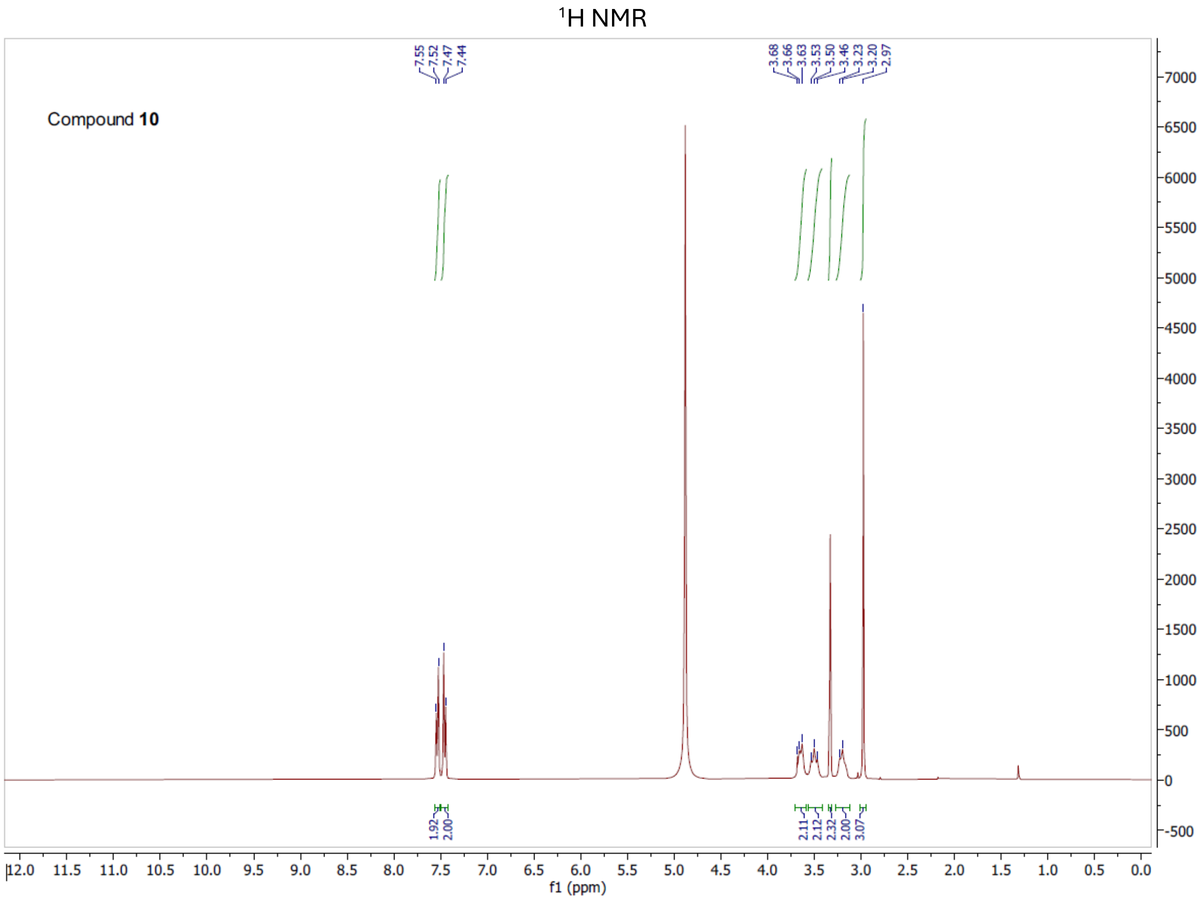

¹H NMR
Compound 10
7.55
7.52
7.47
7.44
3.68
3.66
3.63
3.53
3.50
3.46
3.23
3.20
2.97
1.92
2.00
2.11
2.12
2.32
2.00
3.07
f1 (ppm)

ESI

Formula Predictor Report

Printed at 04.03.2026 11:55:36

Formula Predictor Result	**C14 H18 N7 Cl**
Mass	320.138377009
Error Margin	10 ppm
DBE Range	Not Used
Electron Ions	Both configurations
HC Ratio	Not Used
Nitrogen Rule	Used

#	Score	Pred. (M)	Pred. m/z	Meas. m/z	Diff. (mDa)	Formulae (M)	Ion	Diff. (ppm)	Iso Score	DBE
1	99.52	319.13122	320.13850	320.13838	-0.12	C14 H18 N7 Cl	[M+H]+	-0.377	99.47	9.0

Event#: 1 MS(E+) Ret. Time : [2.740] Scan# : [412] Correction: Success 1.09e4

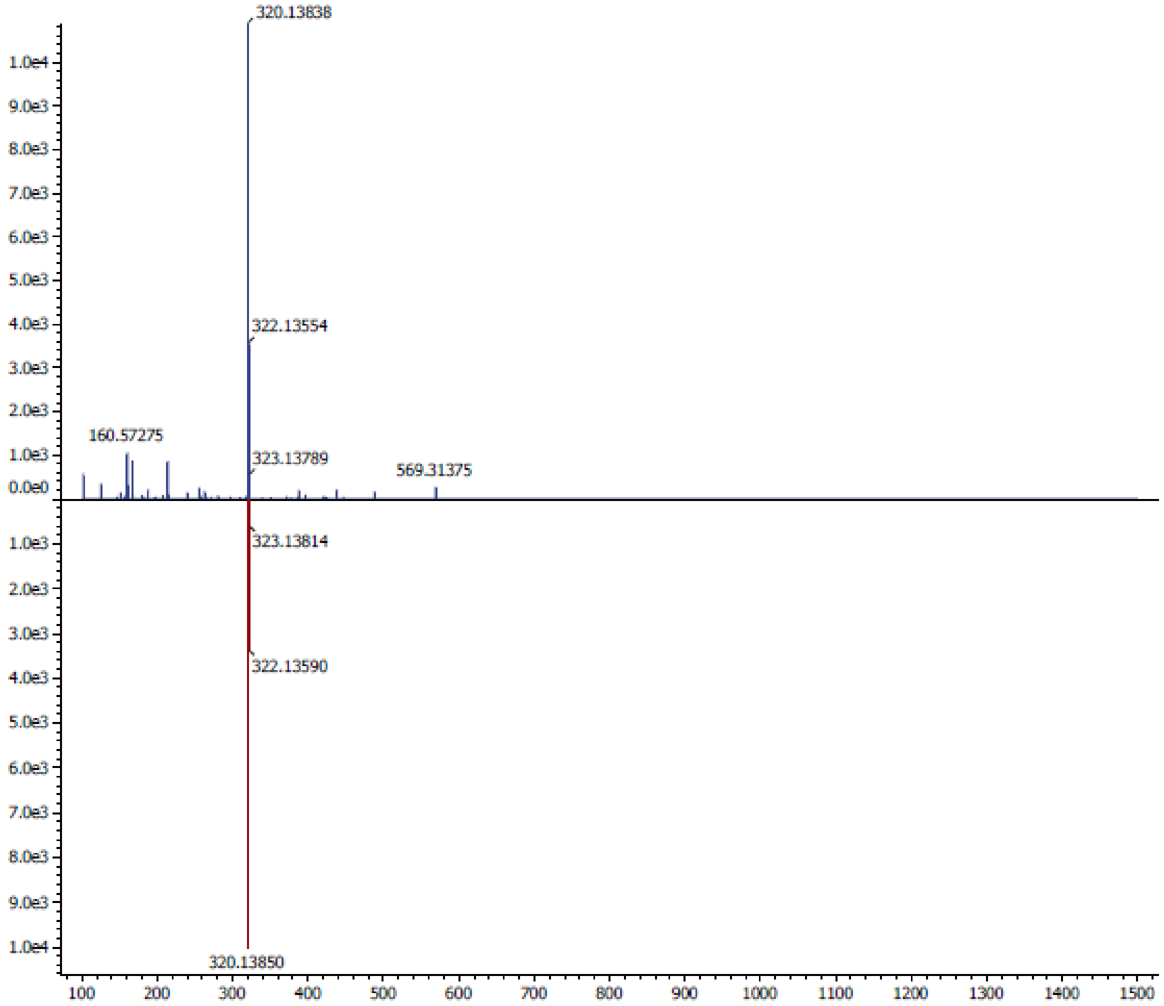

ts([C14 H18 N7 Cl+H]+) 1.00e4

^1H NMR

Compound **11**

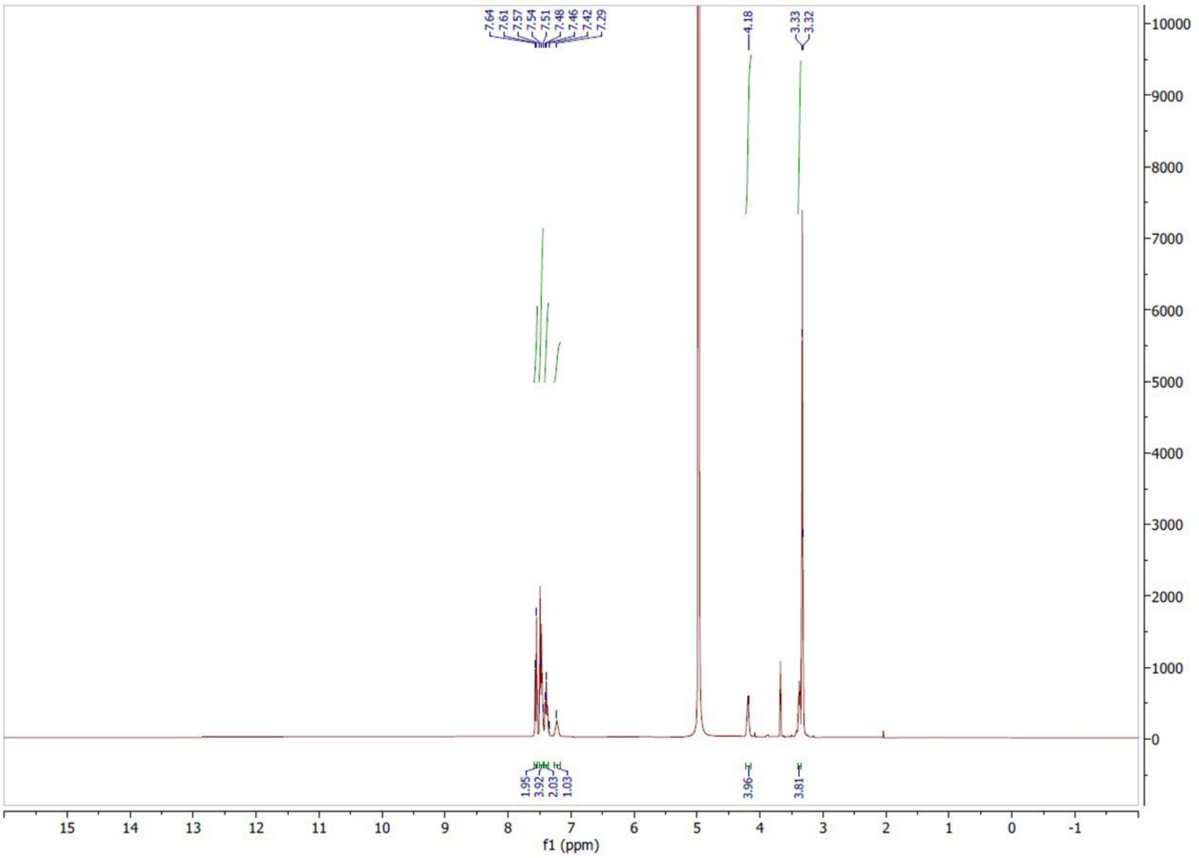

ESI

Formula Predictor Report

Printed at 04.03.2026 13:15:01

Formula Predictor Result	**C19 H20 N7 Cl**
Mass	382.154039461
Error Margin	10 ppm
DBE Range	Not Used
Electron Ions	Both configurations
HC Ratio	Not Used
Nitrogen Rule	Used

#	Score	Pred. (M)	Pred. m/z	Meas. m/z	Diff. (mDa)	Formulae (M)	Ion	Diff. (ppm)	Iso Score	DBE
1	99.44	381.14687	382.15415	382.15404	-0.11	C19 H20 N7 Cl	[M+H]+	-0.284	99.38	13.0

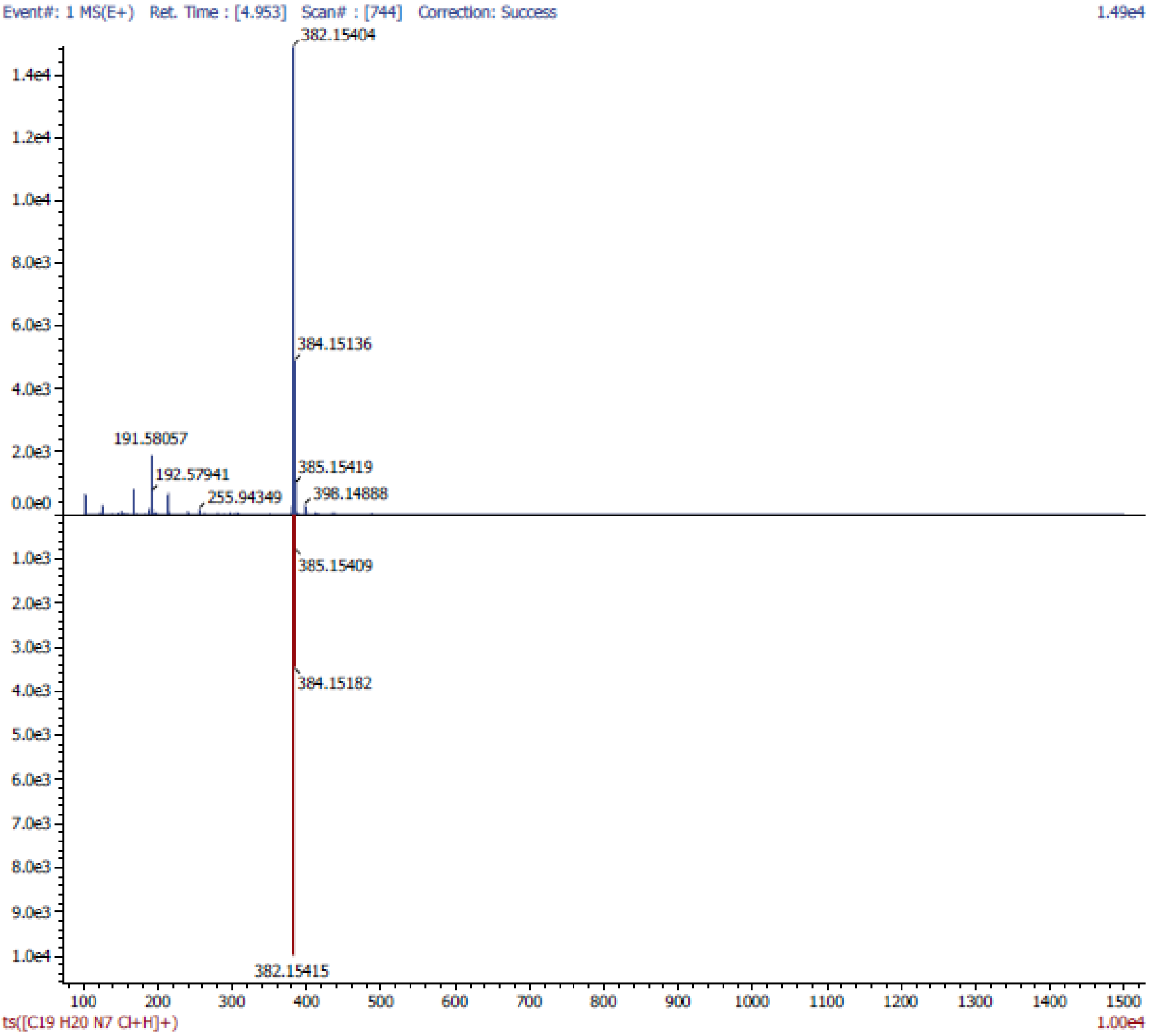

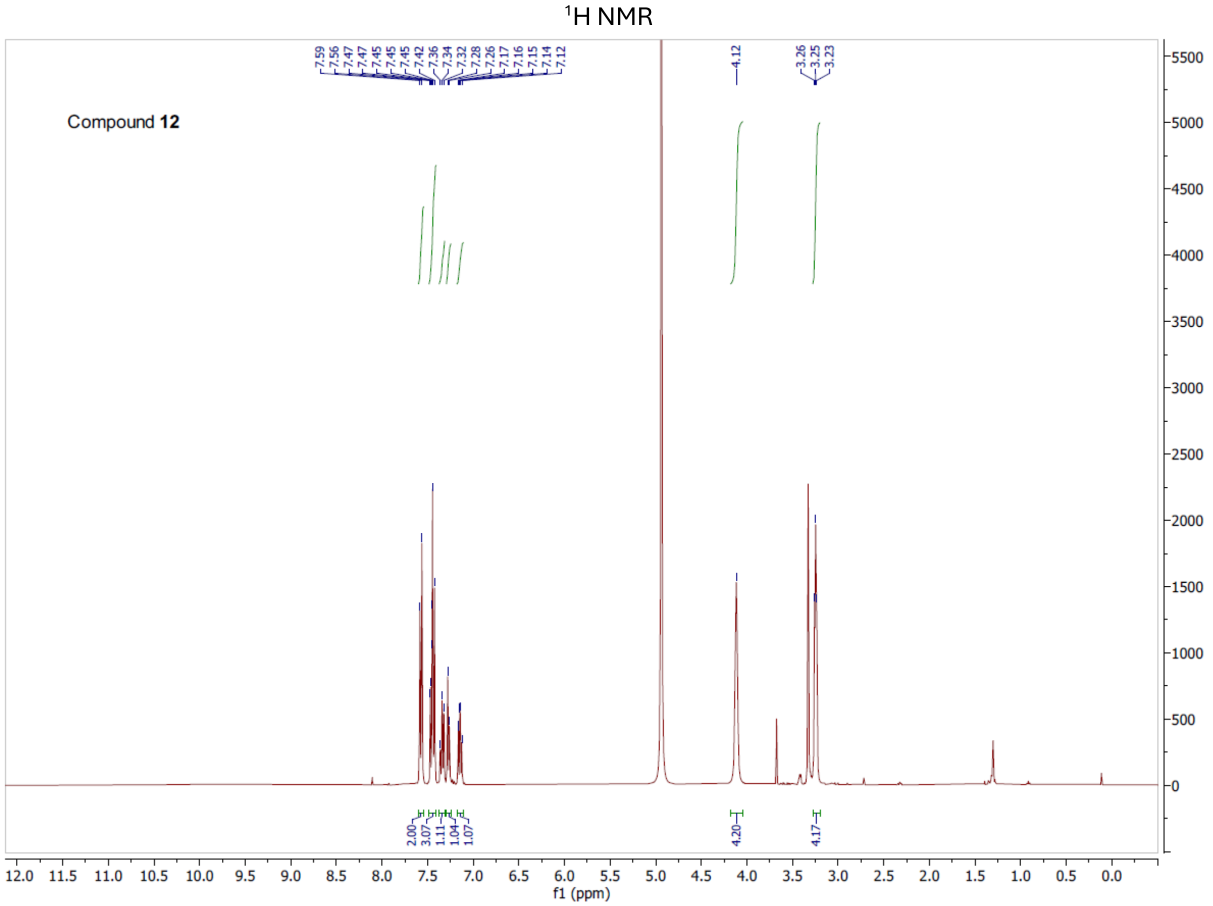

¹H NMR
Compound 12
f1 (ppm)

ESI

Formula Predictor Report

Printed at 08.03.2026 13:54:55

Formula Predictor Result	**C19 H19 N7 Cl2**
Mass	416.1147
Error Margin	10 ppm
DBE Range	Not Used
Electron Ions	Both configurations
HC Ratio	Not Used
Nitrogen Rule	Used

#	Score	Pred. (M)	Pred. m/z	Meas. m/z	Diff. (mDa)	Formulae (M)	Ion	Diff. (ppm)	Iso Score	DBE
1	85.59	415.10790	416.11518	416.11470	-0.48	C19 H19 N7 Cl2	[M+H]+	-1.143	95.09	13.0

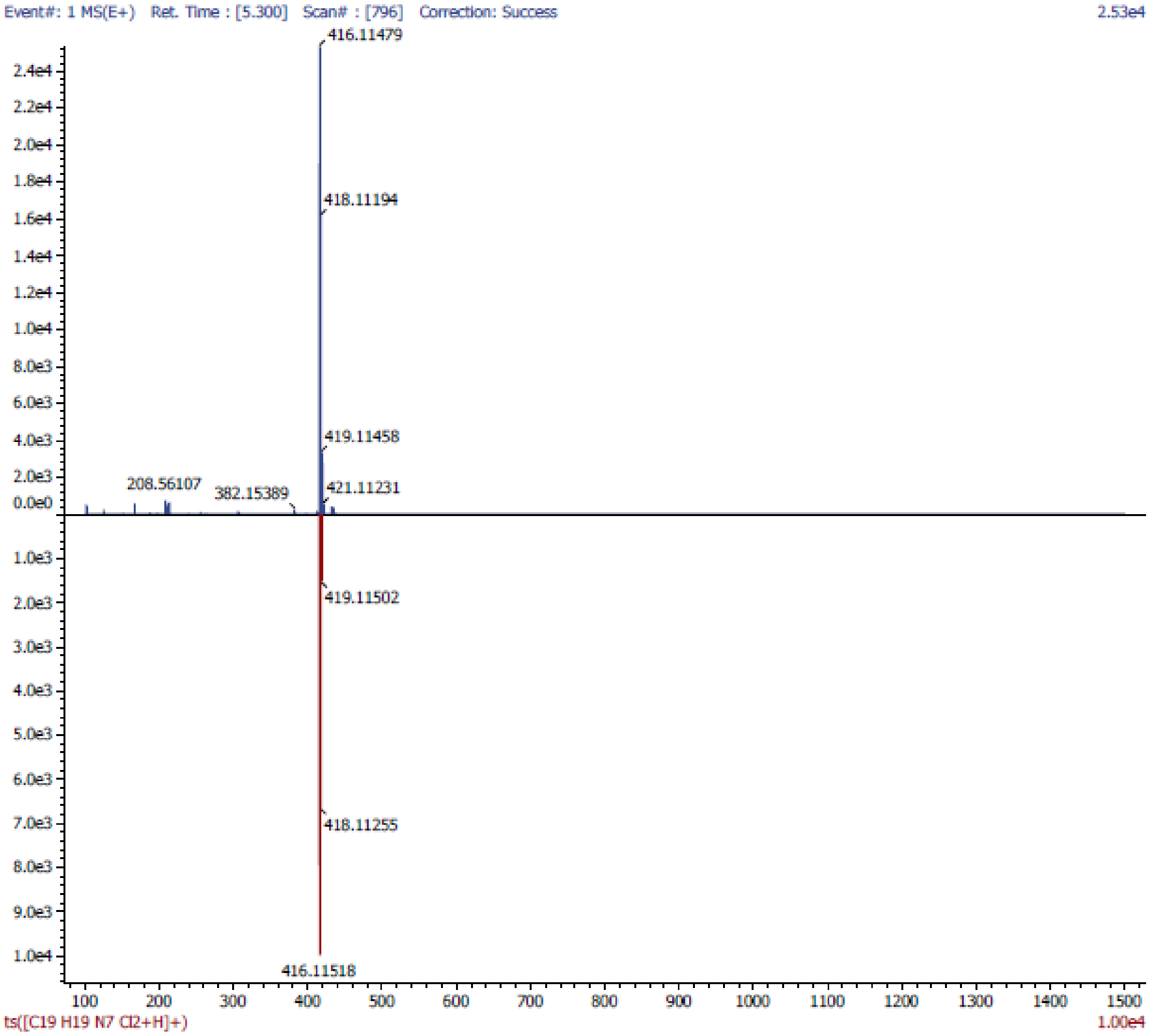

^{1}H NMR

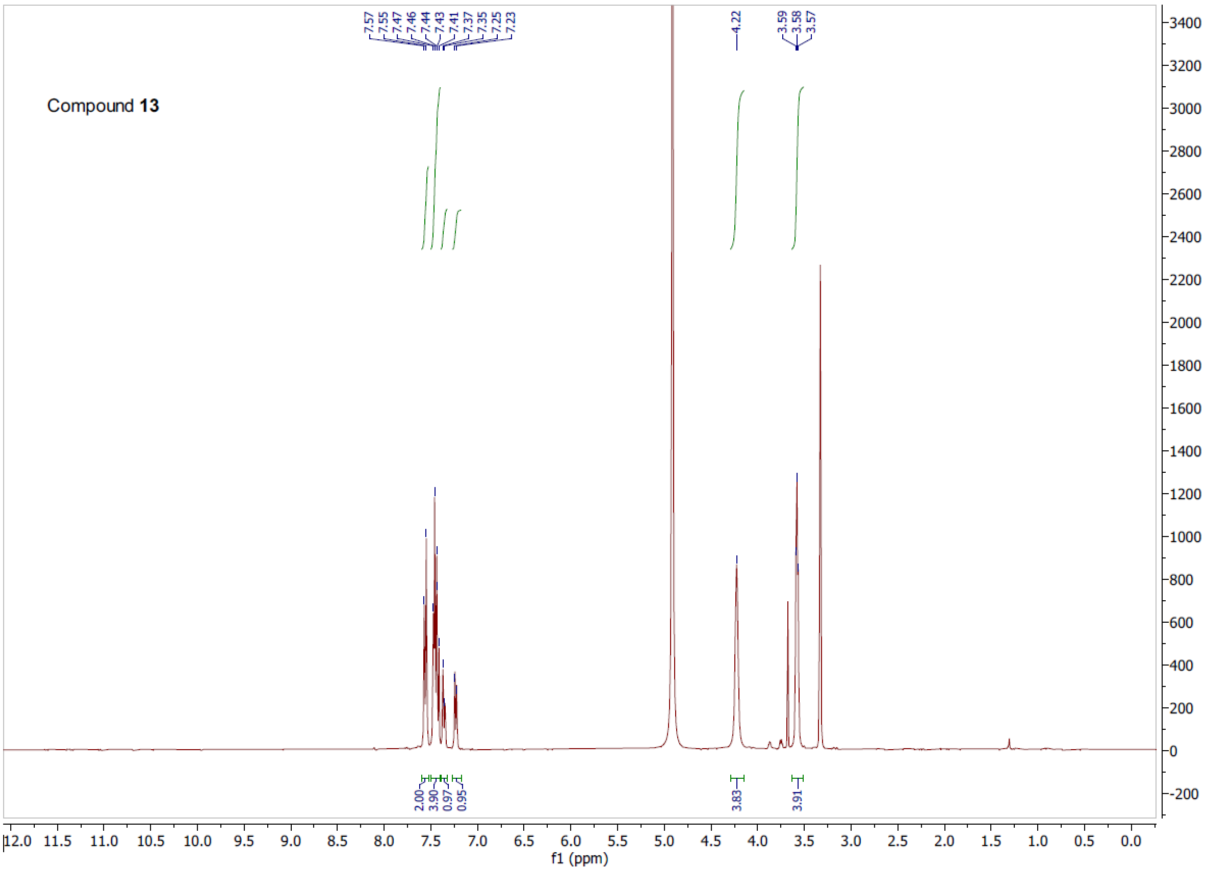

Compound **13**
7.57
7.55
7.47
7.46
7.44
7.43
7.41
7.37
7.35
7.25
7.23
4.22
3.59
3.58
3.57
2.00
3.90
0.97
0.95
3.83
3.91
f1 (ppm)

Formula Predictor Report

Printed at 04.03.2026 13:12:32

Formula Predictor Result	**C19 H19 N7 Cl2**
Mass	416.115
Error Margin	10 ppm
DBE Range	Not Used
Electron Ions	Both configurations
HC Ratio	Not Used
Nitrogen Rule	Used

#	Score	Pred. (M)	Pred. m/z	Meas. m/z	Diff. (mDa)	Formulae (M)	Ion	Diff. (ppm)	Iso Score	DBE
1	97.01	415.10790	416.11518	416.11500	-0.18	C19 H19 N7 Cl2	[M+H]+	-0.422	96.68	13.0

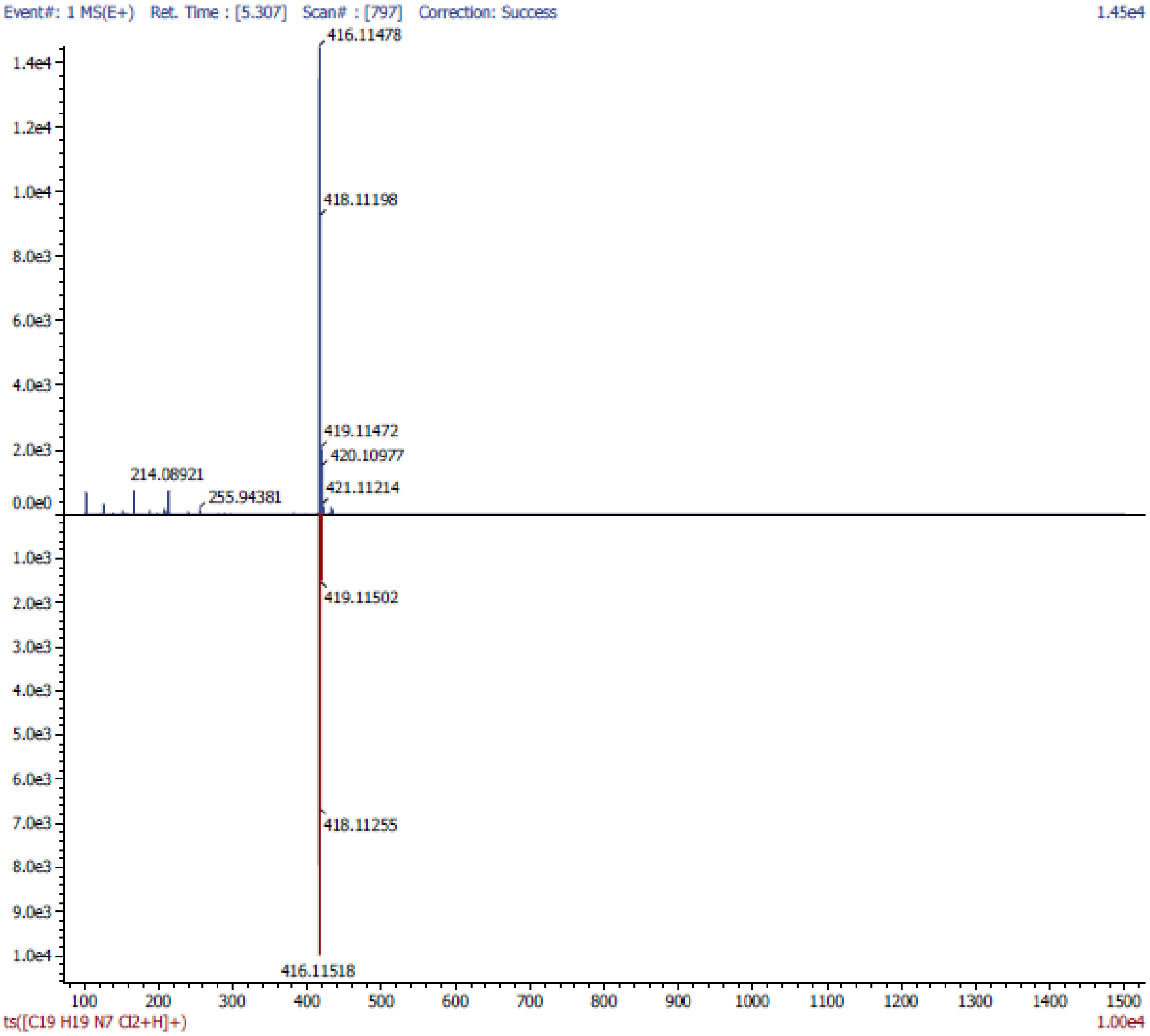

1H NMR

Compound **14**

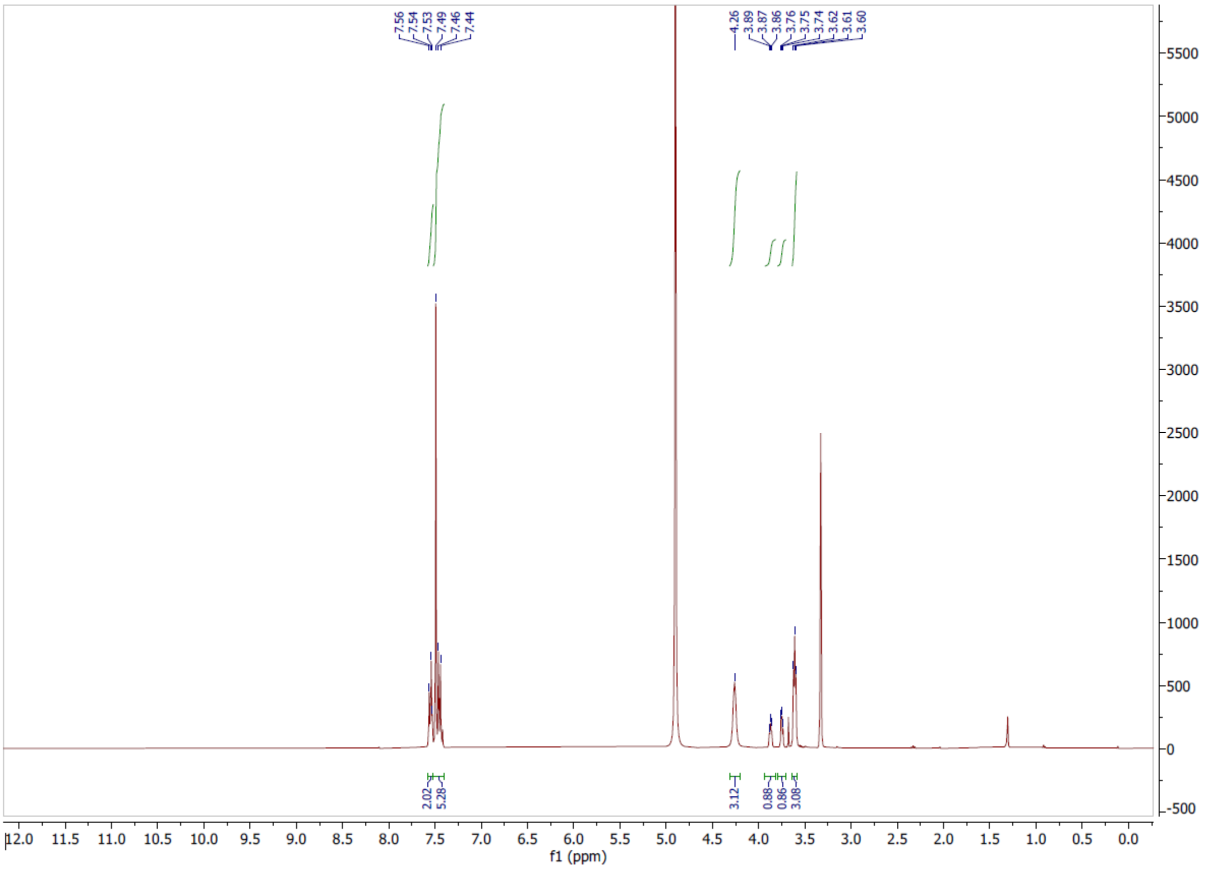

ESI

Formula Predictor Report

Printed at 08.03.2026 13:23:59

Formula Predictor Result	**C19 H19 N7 Cl2**
Mass	416.115
Error Margin	10 ppm
DBE Range	Not Used
Electron Ions	Both configurations
HC Ratio	Not Used
Nitrogen Rule	Used

#	Score	Pred. (M)	Pred. m/z	Meas. m/z	Diff. (mDa)	Formulae (M)	Ion	Diff. (ppm)	Iso Score	DBE
1	98.85	415.10790	416.11518	416.11500	-0.18	C19 H19 N7 Cl2	[M+H]+	-0.422	98.72	13.0

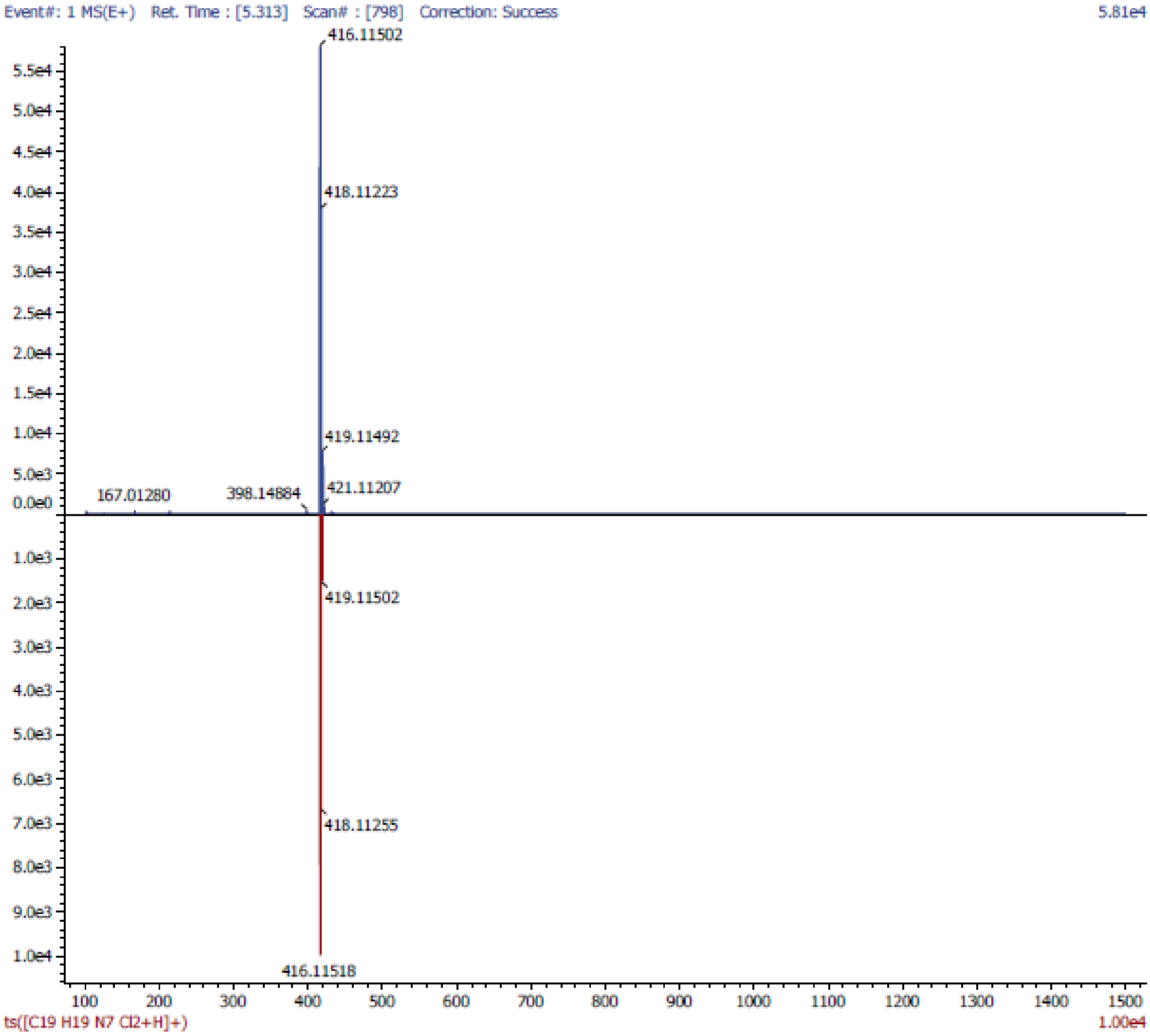

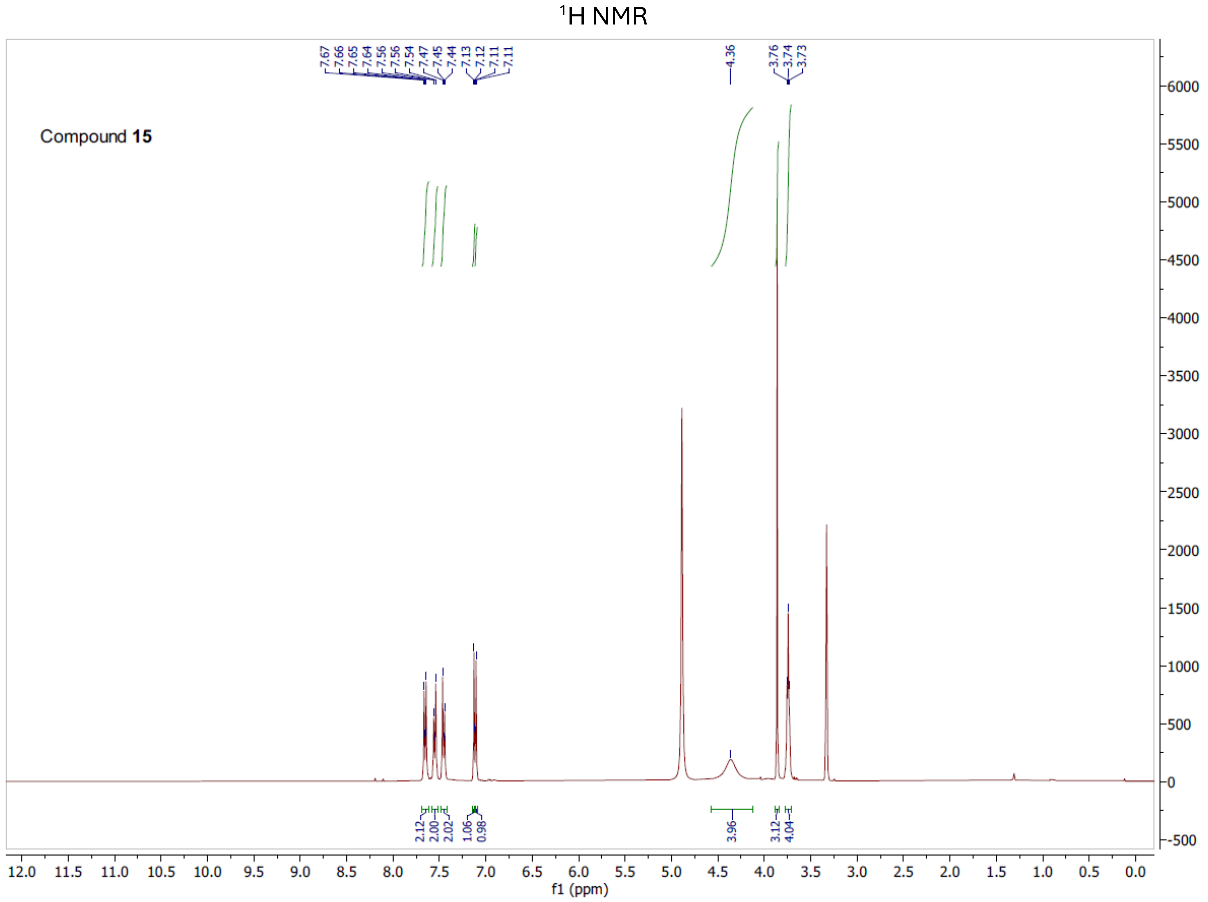

1H NMR
Compound 15
7.67
7.66
7.65
7.64
7.56
7.56
7.54
7.47
7.45
7.44
7.13
7.12
7.11
7.11
4.36
3.76
3.74
3.73
2.12
2.00
2.02
1.06
0.98
3.96
3.12
4.04
f1 (ppm)

ESI

Formula Predictor Report

Printed at 04.03.2026 12:55:09

Formula Predictor Result	**C20 H22 N7 O Cl**
Mass	412.164011606
Error Margin	10 ppm
DBE Range	Not Used
Electron Ions	Both configurations
HC Ratio	Not Used
Nitrogen Rule	Used

#	Score	Pred. (M)	Pred. m/z	Meas. m/z	Diff. (mDa)	Formulae (M)	Ion	Diff. (ppm)	Iso Score	DBE
1	66.12	411.15744	412.16471	412.16401	-0.70	C20 H22 N7 O Cl	[M+H]+	-1.701	62.35	13.0

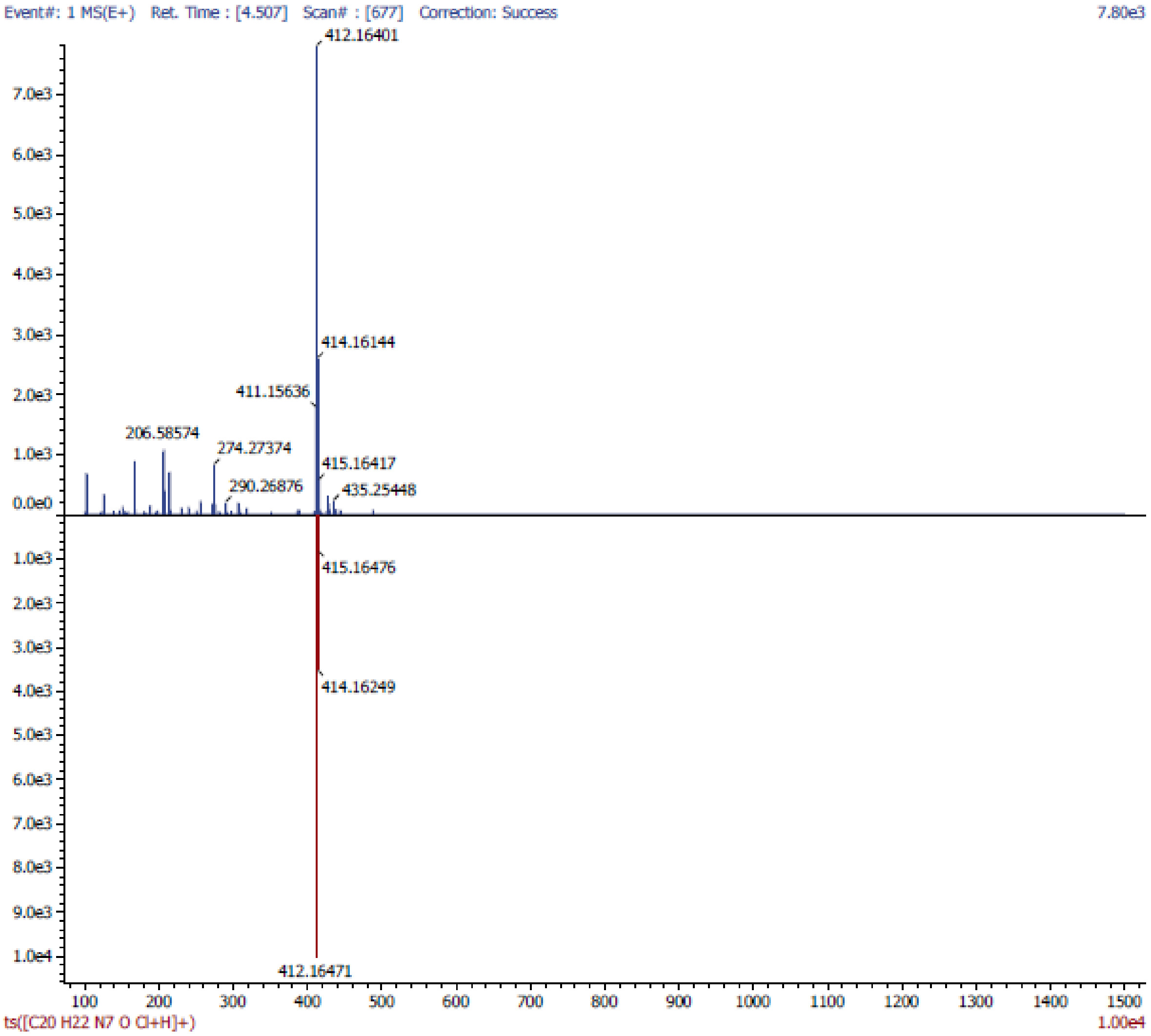

^{1}H NMR

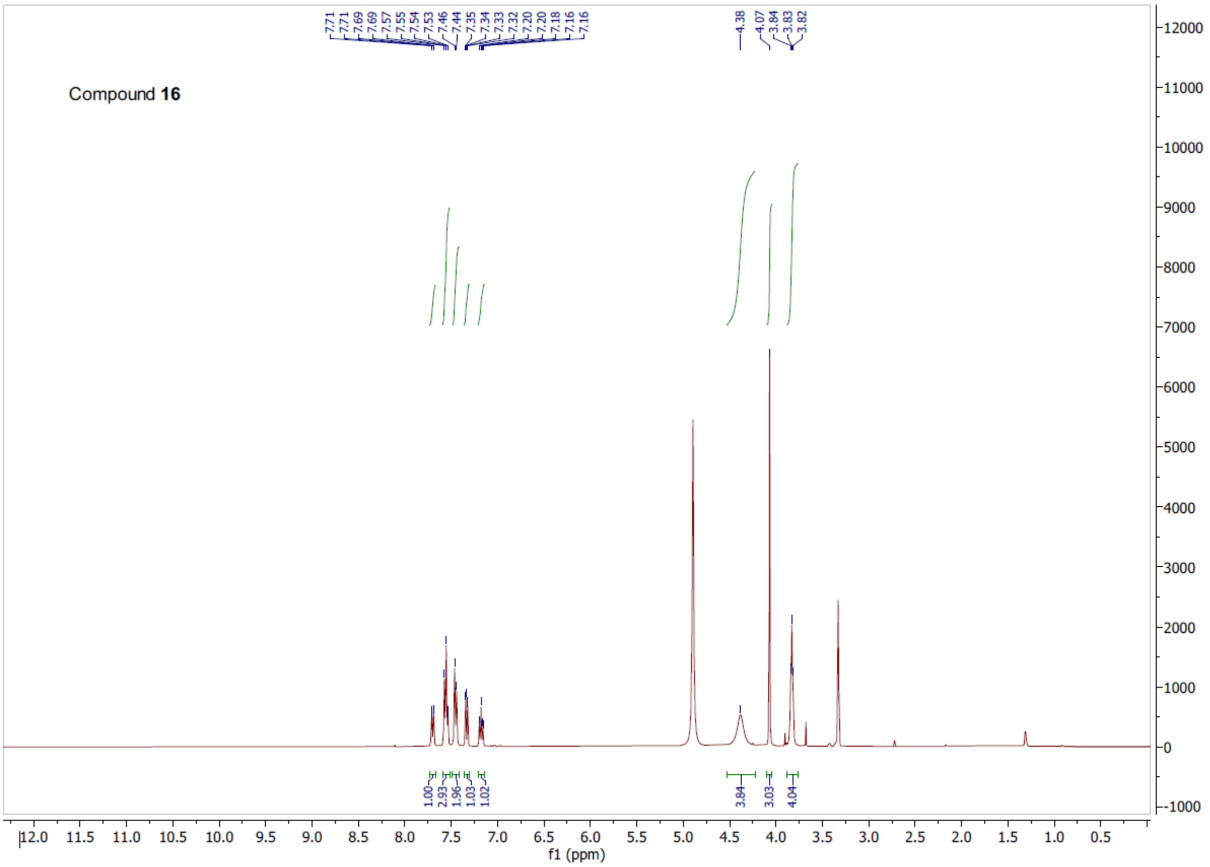

Compound **16**
7.71
7.71
7.69
7.69
7.57
7.55
7.54
7.53
7.46
7.44
7.35
7.34
7.33
7.32
7.20
7.20
7.18
7.16
7.16
4.38
4.07
3.84
3.83
3.82
1.00
2.93
1.96
1.03
1.02
3.84
3.03
4.04
12.0
11.5
11.0
10.5
10.0
9.5
9.0
8.5
8.0
7.5
7.0
6.5
6.0
5.5
5.0
4.5
4.0
3.5
3.0
2.5
2.0
1.5
1.0
0.5
f1 (ppm)
12000
11000
10000
9000
8000
7000
6000
5000
4000
3000
2000
1000
0
-1000

ESI

Formula Predictor Report

Printed at 08.03.2026 13:12:59

Formula Predictor Result	**C20 H22 N7 O Cl**
Mass	412.164
Error Margin	10 ppm
DBE Range	Not Used
Electron Ions	Both configurations
HC Ratio	Not Used
Nitrogen Rule	Used

#	Score	Pred. (M)	Pred. m/z	Meas. m/z	Diff. (mDa)	Formulae (M)	Ion	Diff. (ppm)	Iso Score	DBE
1	97.97	411.15744	412.16471	412.16400	-0.71	C20 H22 N7 O Cl	[M+H]+	-1.729	97.74	13.0

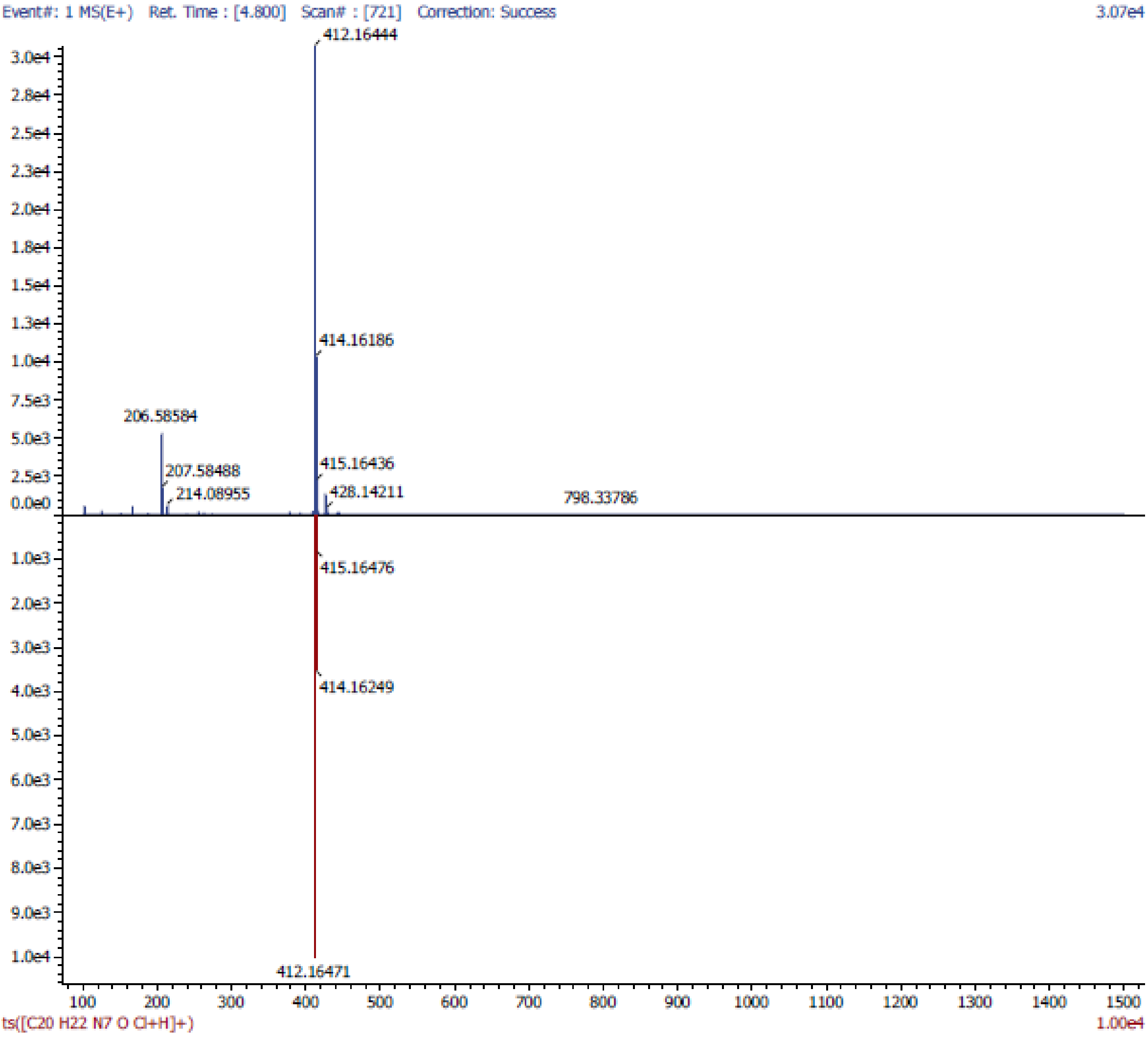

^{1}H NMR

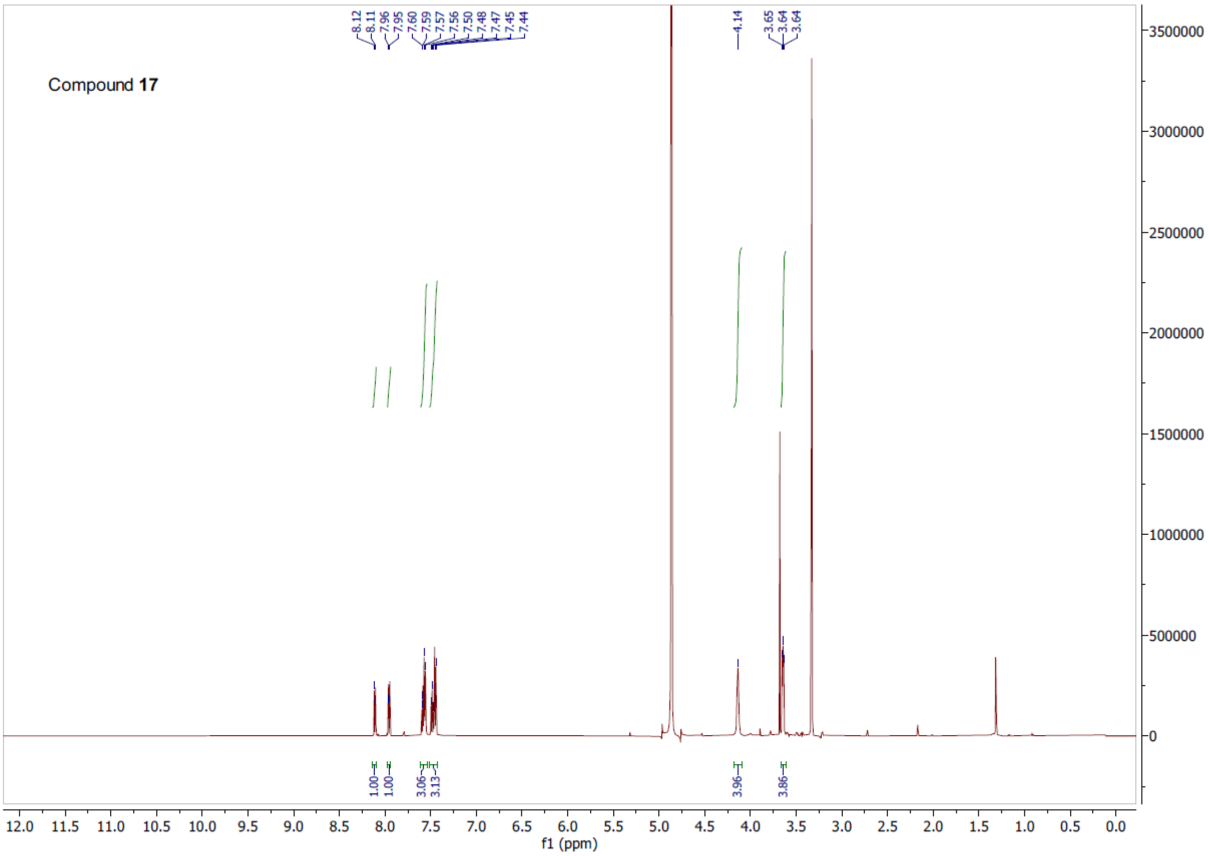
Compound 17
8.12
8.11
7.96
7.95
7.60
7.59
7.57
7.56
7.50
7.48
7.47
7.45
7.44
4.14
3.65
3.64
3.64
1.00
1.00
3.06
3.13
3.96
3.86
f1 (ppm)

Formula Predictor Report

Printed at 08.03.2026 14:14:03

Formula Predictor Result	**C20 H19 N8 S Cl**
Mass	439.121
Error Margin	10 ppm
DBE Range	Not Used
Electron Ions	Both configurations
HC Ratio	Not Used
Nitrogen Rule	Used

#	Score	Pred. (M)	Pred. m/z	Meas. m/z	Diff. (mDa)	Formulae (M)	Ion	Diff. (ppm)	Iso Score	DBE
1	98.87	438.11419	439.12147	439.12100	-0.47	C20 H19 N8 S Cl	[M+H]+	-1.066	98.74	15.0

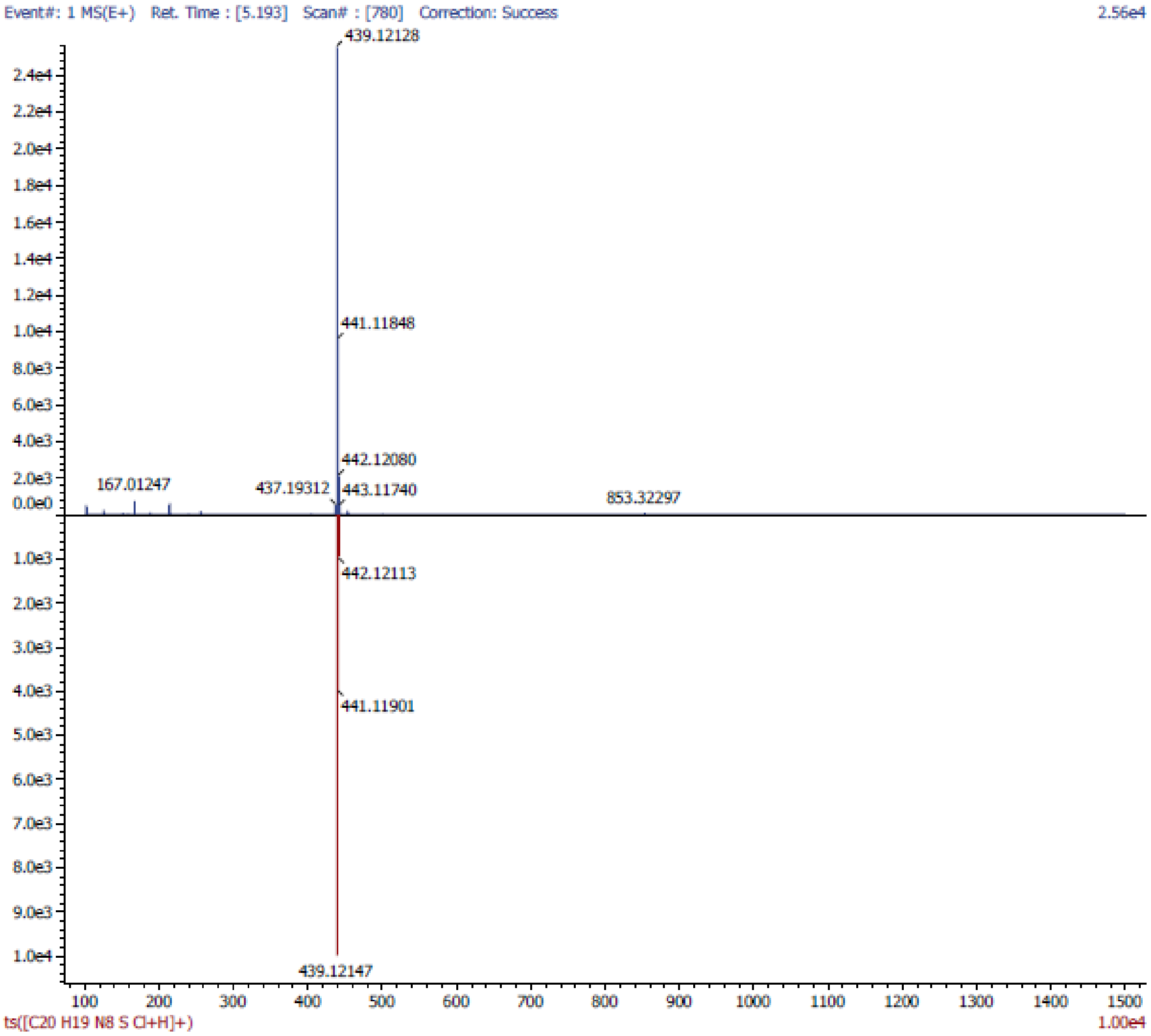

Supplement: Supplementary file 1 [file cimb-48-00319-s001.zip › cimb-4180990-supplementary.pdf]
